# Zinc Finger Protein-Based Prognostic Signature Predicts Survival in Lung Adenocarcinoma

**DOI:** 10.3390/cancers17132203

**Published:** 2025-06-30

**Authors:** Lihui Yu, Yahui Zhou, Jingyu Chen

**Affiliations:** 1 Wuxi School of Medicine, Jiangnan University, Wuxi 214122, China; 2 Department of Neonatology, Affiliated Children’s Hospital of Jiangnan University, Wuxi Children’s Hospital, Wuxi 214023, China; 3 Wuxi Lung Transplant Center, Wuxi People’s Hospital Affiliated to Nanjing Medical University, Wuxi 214023, China

**Keywords:** lung adenocarcinoma, gene signature, zinc finger proteins, tumor immune microenvironment, biomarker

## Abstract

**Background**: Zinc finger proteins (ZNFs), functioning as pervasive transcriptional modulators, serve as pivotal mediators of tumorigenesis and malignant advancement. However, the mechanistic contributions of these epigenetic orchestrators to lung adenocarcinoma pathogenesis remain incompletely characterized. **Methods**: To elucidate zinc finger proteins’ biological significance in lung adenocarcinoma (LUAD) pathogenesis, we first extracted relevant transcriptional data from TCGA. After preliminary screening with univariate Cox regression, a LASSO algorithm was applied to optimize the risk score model, incorporating key zinc finger protein markers. For independent validation, we accessed GEO dataset GSE68465, applying identical analytical protocols to confirm model generalizability. We performed multivariable Cox regression to identify independent predictors of clinical outcomes after adjusting for confounding variables. Cell-based validation included (1) comparative analysis of zinc finger protein expression across LUAD/normal cell models and (2) technical verification using standardized qRT-PCR protocols. **Results**: Following rigorous bioinformatics screening comprising differential expression and survival analysis, the final 21-zinc finger protein cohort was selected for risk score algorithm development aimed at clinical outcome prediction. Stratification based on computed risk scores revealed markedly superior survival outcomes in the low-risk cohort compared to high-risk patients. Comparative analysis revealed overall concordance in the transcriptional profiles of eight ZNFs (|coef| > 0.1) across experimental cell systems and TCGA datasets. **Conclusions**: Collectively, the prognostic framework incorporating zinc finger proteins demonstrates biomarker utility in lung adenocarcinoma survival prediction, while offering novel avenues for molecular target discovery in therapeutic strategies against this malignancy.

## Simple Summary

Zinc finger proteins (ZNFs) are crucial transcriptional regulators in cancer progression, yet their functional roles in lung adenocarcinoma (LUAD) pathogenesis require systematic characterization. This study aimed to develop a ZNF-based prognostic model for LUAD outcome prediction. Transcriptomic data from TCGA were analyzed via univariate Cox and LASSO regression to construct a risk score model. External validation used GEO dataset GSE68465 with identical protocols. Multivariable Cox regression controlled confounders. Experimental validation included: (1) ZNF expression profiling in LUAD/normal cells; (2) qRT-PCR technical verification. Key Results: 1. A 21-ZNF signature stratifies LUAD patients into distinct risk cohorts. 2. Low-risk patients exhibit significantly prolonged survival. 3. Eight core ZNFs (|coef| > 0.1) show consistent dysregulation in cell models and clinical datasets. The ZNF-derived prognostic framework provides a clinically applicable biomarker for LUAD survival prediction. Its integration into precision oncology may accelerate therapeutic target discovery, ultimately improving patient outcomes through personalized risk management.

## 1. Introduction

As the second most prevalent malignancy globally, lung cancer represents a predominant contributor to cancer mortality [1]. Lung adenocarcinoma, constituting nearly half of pulmonary malignancies, predominates among histopathological subtypes [2]. Current therapeutic strategies for LUAD integrate three principal modalities: tumor excision, molecularly targeted interventions, and immune-modulatory approaches [3]; though these advancements have ameliorated clinical outcomes, survival benefits remain limited [4]. Notably, the development of acquired resistance mechanisms against molecular-targeted agents and immune checkpoint inhibitors poses a significant barrier to durable therapeutic responses [5,6]. The discovery of innovative biomarkers could substantially enhance prognostic capabilities and therapeutic stratification for LUAD patients undergoing immunotherapeutic regimens.

Zinc finger proteins, constituting the most abundant transcriptional regulators in humans, exhibit structural polymorphism in DNA-binding domains that critically govern genomic regulation [7]. Structural phylogeny analysis delineates eight principal ZNF superfamilies according to zinc-coordination patterns: Gag knuckle, C2H2-like domains, short zinc-binding loops, Treble clef motifs, zinc ribbon structures, Zn2/Cys6 clusters, TAZ2-like domains, and Metallothionein variants [7,8,9]. This structural heterogeneity enables ZNFs to orchestrate multifaceted biological processes, including apoptotic signaling modulation, metabolic reprogramming, and autophagic flux regulation [10]. Existing studies have indicated a close association between ZNF and the development of malignant tumors [11]. For example, ZNF714 exhibits oncogenic addiction in NSCLC through chromatin remodeling-mediated proto-oncogene activation [12]; ZNF233 silencing attenuates HCC progression via cell cycle arrest induction [13]; ZNF185 exerts tumor-suppressive effects in LUAD by antagonizing AKT/GSK3β signaling axis [14].

As previously noted, despite numerous investigations into zinc finger protein and non-small-cell lung cancer, research concerning the diagnosis and prognosis of lung cancer associated with zinc finger protein genes remains scarce, underscoring the imperative for systematic exploration of such therapeutic targets.

## 2. Materials and Methods

### 2.1. Data Acquisition

This investigation utilized LUAD patient datasets (RNA-seq profiles and clinical annotations) retrieved through the TCGA portal (https://portal.gdc.cancer.gov/, accessed on 25 June 2025). Bioinformatic data curation was performed using TCGAbiolinks for batch-effect-adjusted mRNA expression matrices and harmonized clinical covariate extraction. Core zinc finger gene information (1555 genes; Supplementary Table S1) originated in the HUGO Gene Nomenclature Committee (HGNC) database, with consolidation through existing evidence [15]. External validation was conducted using the GSE68465 cohort (n = 442) accessed via NCBI’s GEO repository (GSE68465, https://www.ncbi.nlm.nih.gov/geo/, accessed on 25 June 2025). This study exclusively utilized open-access datasets from established repositories, thereby precluding requirements for in vivo experimentation or institutional ethics review.

### 2.2. Identification of Prognostic Associated Zinc Finger Protein Genes

Initially, the acquired data underwent preprocessing: (1) Genes exhibiting zero expression across all samples were excluded. (2) For genes with multiple entries, the average value was calculated. (3) All data underwent standardization via log-transformation. Subsequently, differentially expressed genes (DEGs) were screened using the following criteria: |LogFC| > 1, Fdr < 0.05. Lastly, DEGs were overlapped with ZNF genes, yielding the differentially expressed ZNF genes. During model training, prognostic ZNF gene signatures were identified through a two-stage selection process: (1) initial filtration via Cox proportional hazards modeling (*p* < 0.05), and (2) feature dimension reduction using LASSO (Least Absolute Shrinkage and Selection Operator) penalized-regression. The LASSO penalty parameter (λ) was determined as lambda. min (λ = 0.0161), corresponding to the minimum cross-validated error. The final prognostic signature value (RSV) was derived from the linear combination: RSV = Σ(β_i × RNA_i), where β denotes regression coefficients and RNA_i represents normalized expression of selected transcripts.

### 2.3. Construction and Calibration of a Prognostic Nomogram

To investigate the prognostic value of zinc finger protein genes in lung adenocarcinoma, we analyzed a training cohort comprising 507 cases with complete survival follow-up. Candidate ZNFs were initially filtered through univariate analysis and LASSO regularization, followed by multivariate Cox proportional hazards modeling to determine the independent prognostic significance of risk scores combined with clinicopathological variables. We established a prognostic nomogram incorporating both molecular risk stratification and clinical parameters, enabling dynamic prediction of overall survival rates at 1-, 3-, and 5-year timepoints. Model performance was validated through time-dependent receiver operating characteristic (ROC) analysis for discriminative capacity evaluation, complemented by calibration curve assessment to quantify prediction consistency. Computational implementation leveraged the “TimeROC” package for ROC curve generation and the “rms” package for nomogram development and cross-validation.

### 2.4. Differential Genes: PPI and Functional Enrichment Analysis

Protein-protein interaction (PPI) networks of transcriptionally discordant genes were constructed via the STRING platform (https://string-db.org/). Functional characterization of transcriptional variants was performed with the “clusterProfiler” RStudio version 4.4.1 for GO term and KEGG pathway analysis, while enriched pathways were visualized using “ggplot2” package. Pathway activity divergence between risk-stratified subgroups was quantified through GSVA algorithm implementation on transcriptionally altered genes.

### 2.5. Tumor Microenvironment Analysis

Using the ESTIMATE algorithm, stromal and immune cell infiltration levels in lung adenocarcinoma tissues were quantified through transcriptomic profiling, yielding three microenvironmental indices: immune/stromal/ESTIMATE scores. Subsequent analyses revealed correlations between molecular risk stratification and tumor immune-stromal contexture. The CIBERSORT deconvolution algorithm (https://cibersort.stanford.edu/) was implemented to profile tumor-infiltrating immune cell (TIIC) subtypes and compare immune cell abundance/functional states across risk-stratified subgroups.

### 2.6. Drug Sensitivity Screening

Therapeutic response profiling for LUAD patients was performed through the Genomics of Drug Sensitivity in Cancer (GDSC) database to evaluate efficacy potentials of standard chemotherapy regimens and molecularly targeted agents. Drug response quantification was achieved using the “oncoPredict” computational framework, which estimates half-maximal inhibitory concentrations (IC50) through machine learning-based modeling of pharmacogenomic datasets.

### 2.7. Cell Culture

Human bronchial epithelium normal (BEAS-2B, Cat: SNL-203) and LUAD cellular models were obtained from Procell Life Science & Technology Co., Ltd. (Wuhan, China). The experimental cell panel comprised the following: (1) BEAS-2B as non-malignant control; (2) LUAD models A549 (Cat: SNL-089), H1975 (Cat: SNL-087), and PC9 (Cat: SNL-152). Differential cultivation protocols were implemented: LUAD cells were propagated in RPMI-1640, whereas BEAS-2B utilized DMEM. Standardized supplementation contained 10% FBS and 1% penicillin-streptomycin. Cellular maintenance occurred in humidified incubators with the following physiological parameters: 5% CO_2_ tension, 37 °C core temperature, and normoxic atmosphere.

### 2.8. RNA Extraction and Real-Time PCR

All primers (sequences in Supplementary Table S2) were synthesized by Aikerui Biotech (Changsha, China). Total RNA isolation followed the Beyotime RNA Kit (R0027, Shanghai, China) protocol. qPCR reactions utilized Novizan’s Vazyne SYBR Mix (Nanjing, China) (Q711-02) with standard cycling conditions. Amplification detection used the ABI 7500 System (Thermo Fisher, Waltham, MA, USA).

### 2.9. Statistical Analysis

Quantitative data processing was conducted with GraphPad Prism (version 8.0) and the R software (v4.3.3) for statistical computation. Intergroup comparisons (two-group vs. multi-group) employed unpaired *t*-tests and ANOVA, respectively. The “limma” package conducted differential expression analysis, while “survival” and “survminer” constructed Kaplan-Meier curves. Continuous variables were presented using arithmetic mean with standard deviation. Statistical significance was defined as *p* < 0.05.

## 3. Results

### 3.1. Identification of Differential ZNFs in LUAD

**Figure 1:**
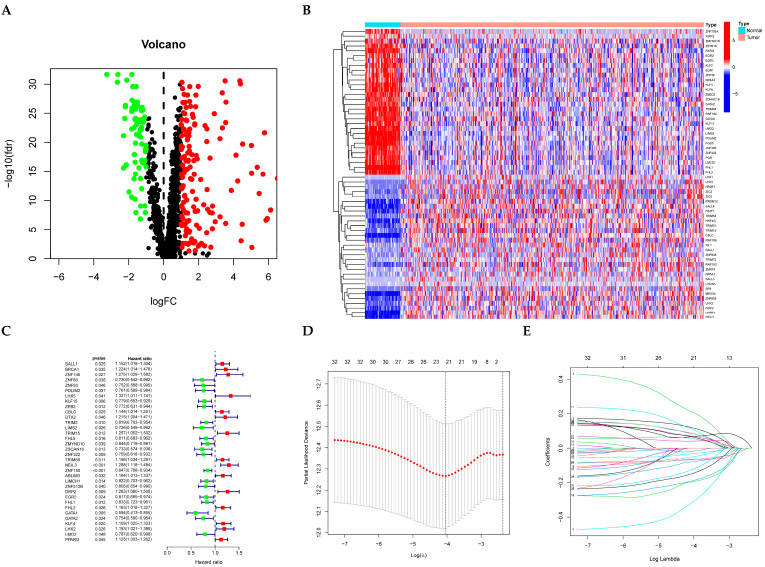
Construction of risk signature in the Validation cohort. Volcano plot (**A**), Red: Significantly upregulated differentially expressed genes (DEGs)Green: Significantly downregulated DEGsBlack: Non-significant genes (adjusted *p* > 0.05), and heatmap (**B**) of 1555 ZNFs in normal and LUAD tissues from training cohort. Univariate cox regression analysis (**C**). LASSO regression analysis: cross-validation error curve. Dashed lines indicate λ_min (**left**) and λ_1se (**right**). (**D**) Coefficient paths of candidate genes. Genes with non-zero coefficients at λ_1se are labeled (**E**).

Comparative transcriptomic analysis via the “limma” package revealed 158 upregulated and 72 downregulated genes in tumor versus normal tissue comparisons (Figure 1A). In this visualization, green dots correspond to down-regulated transcripts, red markers reflect up-regulated counterparts, and black points denote non-significant entities. Significance thresholds included |logFC| > 1 with FDR-adjusted *p* < 0.05. Figure 1B graphically presents clustering patterns of differentially expressed zinc finger protein genes employing agglomerative clustering, hierarchical clustering was employed for gene-level organization (rows), while specimen dimensions (columns) retained initial sequencing.

### 3.2. Construction and Validation of ZNFs-Associated Risk Score Model in LUAD

**Figure 2:**
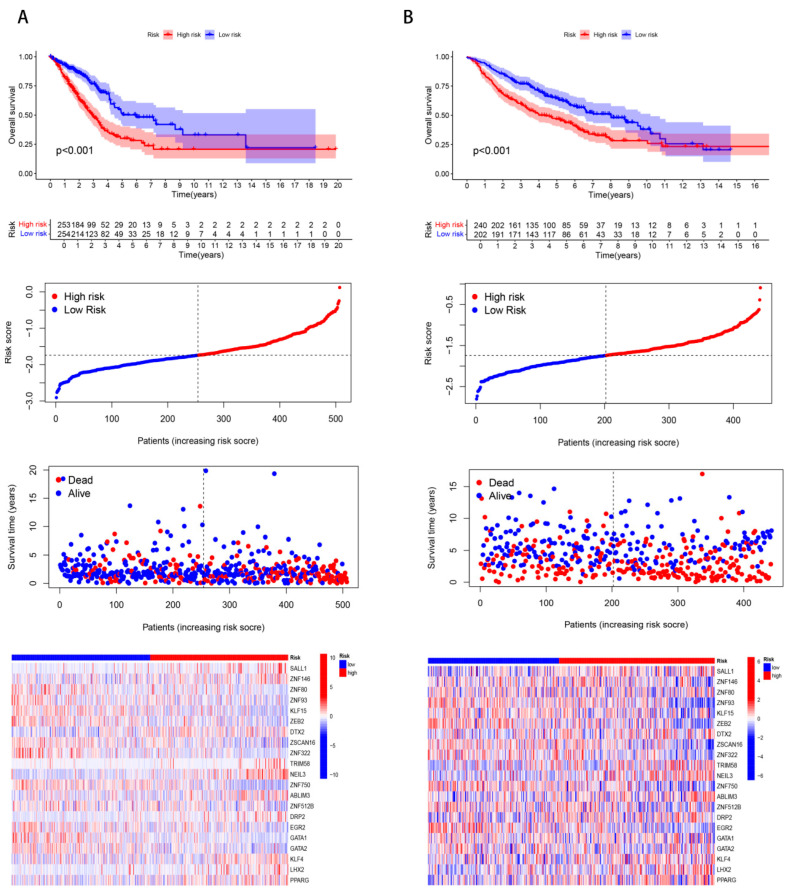
Prognostic analysis of a model based on 21 ZNFs in the training and validation sets. Training set (**A**). Validation set (**B**). From top to bottom, they, respectively, represent the survival status, survival time and risk score distribution in the high- and low-risk groups, as well as the expression heat maps of 21 prognostic genes in the high- and low-risk groups.

Cox proportional hazards regression (Figure 1C) and LASSO regularization modeling (Figure 1D,E) were conducted in LUAD cohorts with comprehensive survival follow-up. Twenty-one ZNF genes (SALL1, ZNF146, ZNF80, ZNF93, KLF15, ZEB2, DTX2, ZSCAN16, ZNF322, TRIM58, NEIL3, ZNF750, ABLIM3, ZNF512B, DRP2, EGR2, GATA1, GATA2, KLF4, LHX2, PPARG) demonstrated significant survival correlations (*p* < 0.05). Risk stratification using median score thresholds categorized patients into high- and low-risk subgroups. Kaplan-Meier curves confirmed superior OS in low-risk versus high-risk cohorts (Figure 2A,B, *p* < 0.001).

### 3.3. Construction and Validation of the Nomogram

**Figure 3:**
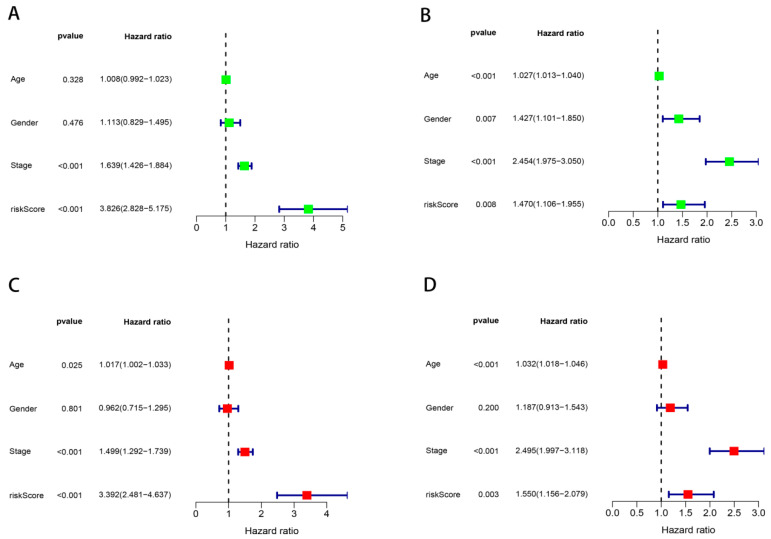
Univariate-multivariate independent prognostic analyses. The green denotes univariate Cox regression, whereas the red indicates multiple Cox regression. Univariate and multivariate independent prognostic analysis in the training cohort (**A**,**C**) and validation cohort (**B**,**D**). Univariate assessment initially screens whether specific factors influence outcomes, while multivariate analysis adjusts for confounder effects following this exploratory step, yielding enhanced reliability.

**Figure 4:**
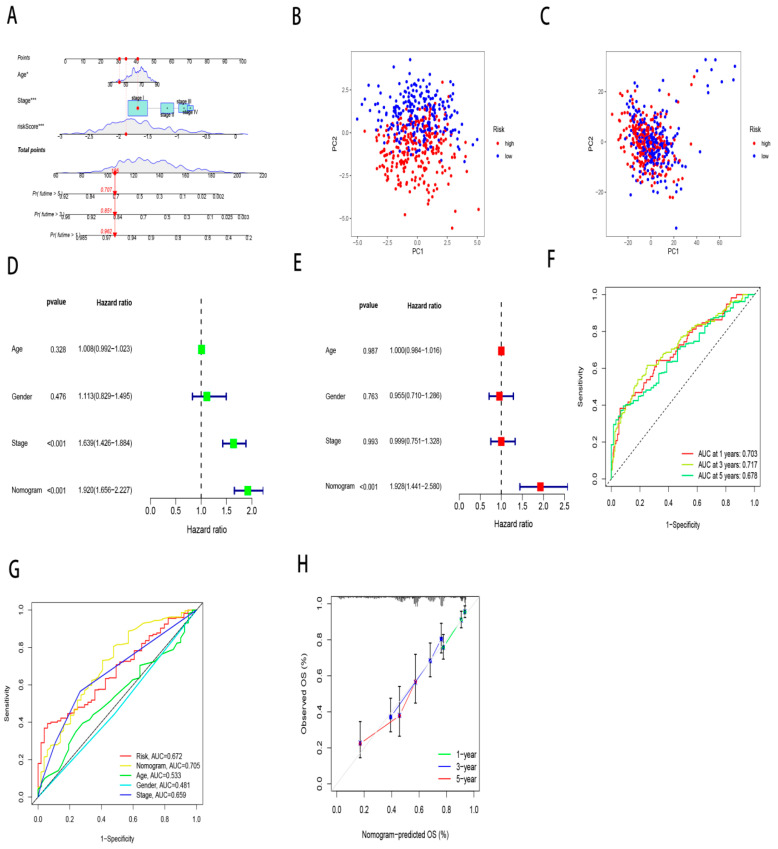
Establishment of a predictive model based on zinc finger proteins. A nomogram integrating risk scores and clinical characteristics for predicting 1-year, 3-year, and 5-year OS (**A**). PCA plot of the nomogram (**B**) and ZNFs (**C**). Univariate (**D**) and multivariate (**E**) analysis of nomograms. (**F**) Time-dependent AUCs for 1-, 3-, and 5-year survival yielded 0.703, 0.717, and 0.678. (**G**) ROC analysis evaluating the nomogram’s power discriminating between risk scores versus clinical variables. (**H**) Demonstrates the calibration curve for the nomogram. * *p* < 0.05, *** *p* < 0.001.

Figure 3A,C demonstrate age, stage and risk score, which are independent prognostic indicators for survival. Validation cohort confirmation appears within the validation cohort (Figure 3B,D). Cox multivariate modeling established clinical stage, age and risk score as independent survival predictors. A prognostic nomogram integrating these variables was formulated to estimate 1-, 3-, 5-year survival probabilities, demonstrating clinical utility (Figure 4A). Elevated model scores predict poorer prognosis. The 1-year survival probability reflects short-term outcomes, the 3-year evaluates medium-term outcomes, while the 5-year guides long-term clinical management. PCA revealed superior risk stratification capacity of the nomogram compared to ZNF-derived scores (Figure 4B,C). Time-dependent ROC analysis confirmed the model’s superior discriminative power for 1-, 3-, 5-year survival prediction (AUC = 0.703,0.717,0.678; Figure 4F), outperforming conventional clinical parameters (Figure 4G). Calibration curves quantitatively assess agreement between forecasted outcomes and observed event frequencies. Figure 4H demonstrates 1-/3-/5-year model projections closely align with empirical event rates (nearly overlapping the 45-degree reference), reflecting robust calibration performance achieved by this prognostic model. This validated nomogram emerged as an independent OS determinant (Figure 4D,E) with critical implications for personalized therapeutic strategies.

### 3.4. PPI and Enrichment Analysis

**Figure 5:**
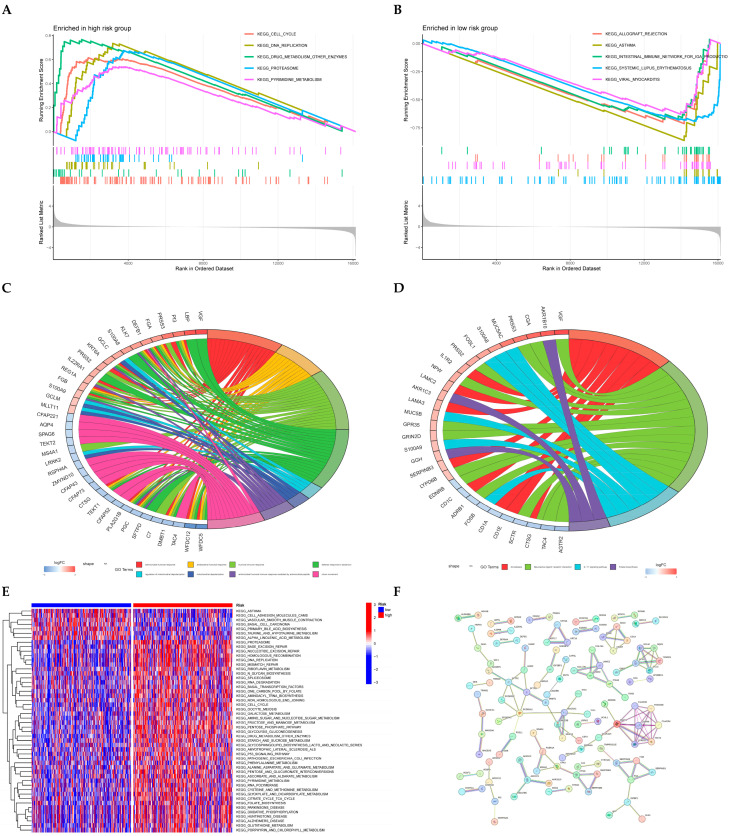
The enrichment analysis of differential genes in different risk groups. GSEA of high-risk group (**A**) and low-risk group (**B**). GO (**C**) and KEGG (**D**) analysis of differentially expressed genes between high- and low-risk groups. GSVA Enrichment Results (**E**). PPI network (**F**): Individual nodes correspond to distinct protein entities. Interconnecting edges signify protein-protein associations. Vertex coloration denotes functional module membership, while edge hue designates association category.

GSEA pathway profiling revealed predominant activation of cell cycle regulation, DNA replication machinery, xenobiotic metabolic enzymes, proteasomal degradation, and pyrimidine biosynthesis in high-risk patients (Figure 5A). Conversely, low-risk cohorts exhibited significant involvement in immune-mediated processes including transplant rejection pathways, asthmatic inflammation, gut IgA immunity networks, systemic autoimmune responses, and viral cardiac pathogenesis (Figure 5B). Functional annotation results from Gene Ontology (GO) and Kyoto Encyclopedia pathways are detailed in Figure 5C,D, respectively. Gene Set Variation Analysis (GSVA; Figure 5E) quantitatively confirmed distinct pathway activation patterns across risk stratifications. Mechanistically, low-risk stratification correlated with asthma-related signaling, CAM-mediated adhesion dynamics, vascular tone modulation, epithelial carcinogenesis pathways, and bile acid synthesis regulation. High-risk phenotypes demonstrated enhanced proteasomal processing, DNA repair mechanisms (base/nucleotide excision, homologous recombination), and replicative machinery activation. The PPI network (Figure 5F) deciphered functional crosstalk between differentially expressed genes underpinning intergroup phenotypic divergence.

### 3.5. Tumor Immune Microenvironment of LUAD

**Figure 6:**
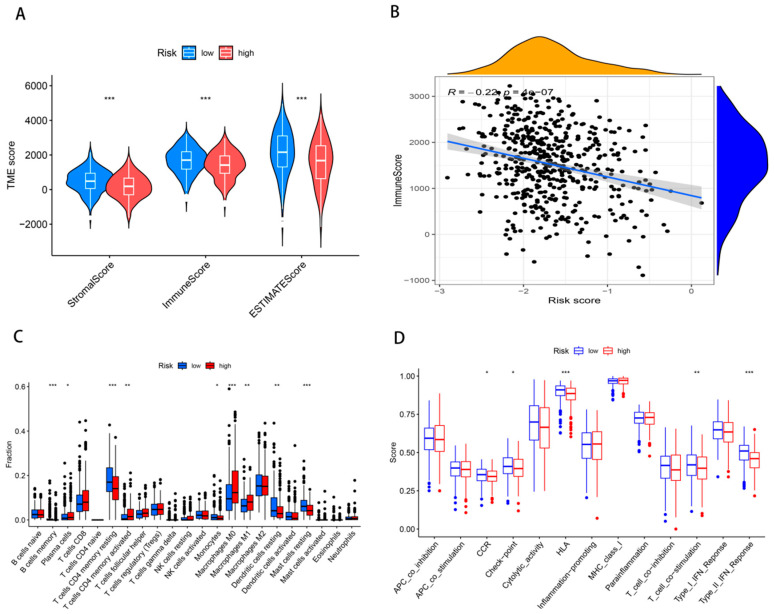
Tumor microenvironment analysis between different risk groups. Differences in ImmuneScore, StromalScore, and ESTIMATEScore between high- and low-risk groups (**A**). Pearson correlation analysis of signature scores and ImmuneScore (**B**). Analysis of tumor-infiltrating cells (**C**) and immune function (**D**) between two groups. * *p* < 0.5, ** *p* < 0.01, *** *p*< 0.001.

Figure 6A demonstrates markedly reduced Immunescore, Stromalscore, and ESTIMATE indices in low-risk versus high-risk cohorts. Pearson’s coefficients revealed an inverse relationship between risk stratification and immune infiltration levels (R = −0.22, *p* < 0.001; Figure 6B). Distinct immune cell composition profiles distinguished the risk stratifications. Low-risk cohorts exhibited elevated memory B cell populations, quiescent CD4+ T lymphocytes, and inactive mastocytes. Conversely, M0-polarized macrophages dominated the high-risk microenvironment (Figure 6C). Functional divergence manifested through HLA-mediated antigen presentation and IFN-γ signaling pathways (Figure 6D), delineating differential immunoregulatory networks across risk categories.

The role of the ZNF-score in antineoplastic drug therapy.

**Figure 7:**
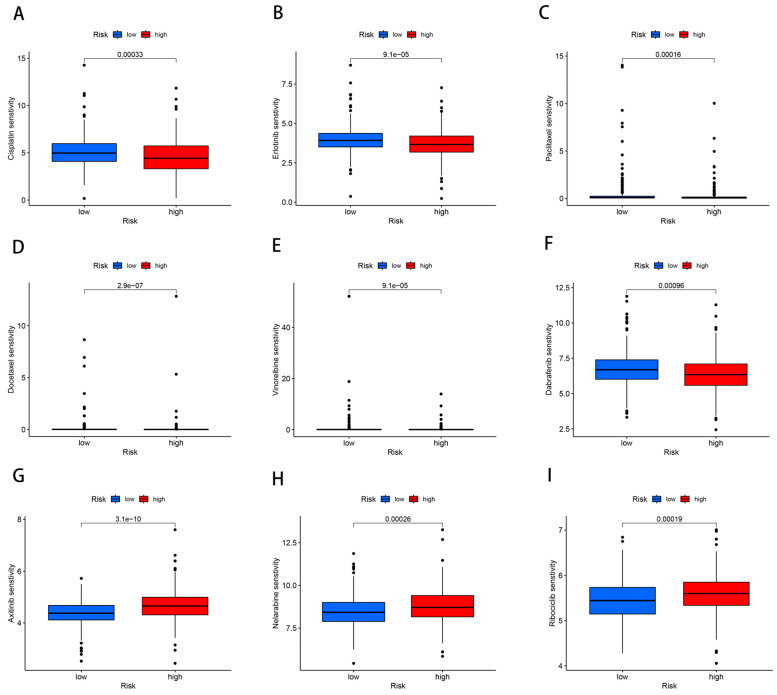
Results of drug sensitivity analysis. Cisplatin (**A**). Erlotinib (**B**). Paclitaxel (**C**). Docetaxel (**D**). Vinorelbine (**E**). Dabrafenib (**F**). Axitinib (**G**). Nelarabine (**H**). Ribociclib (**I**).

Therapeutic responses to standard LUAD chemotherapeutics were evaluated across risk-stratified cohorts. Low-risk patients demonstrated significantly enhanced sensitivity to cisplatin (Figure 7A), erlotinib (Figure 7B), paclitaxel (Figure 7C), docetaxel (Figure 7D), vinorelbine (Figure 7E), and dabrafenib (Figure 7F) (all *p* < 0.001). Conversely, axitinib (Figure 7G), nelarabine (Figure 7H), and ribociclib (Figure 7I) showed preferential sensitivity in high-risk subgroups. The ZNF-based stratification demonstrated predictive capacity for chemotherapy selection and treatment outcome evaluation.

### 3.6. Validation in LUAD Cell Lines

**Figure 8:**
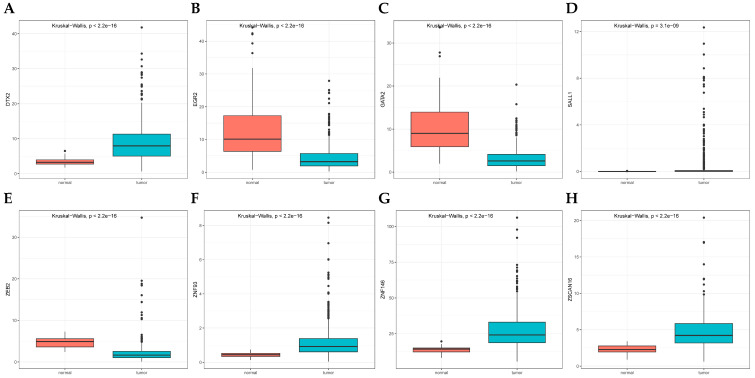
Expression of eight ZNF genes from the validation cohort in LUAD tissue. DTX2 (**A**), SALL1 (**D**), ZNF93 (**F**), ZNF146 (**G**) and ZSCAN16 (**H**) are relatively highly expressed in tumor tissues, whereas EGR2 (**B**), GATA2 (**C**), and ZEB2 (**E**) are relatively less expressed.

**Figure 9:**
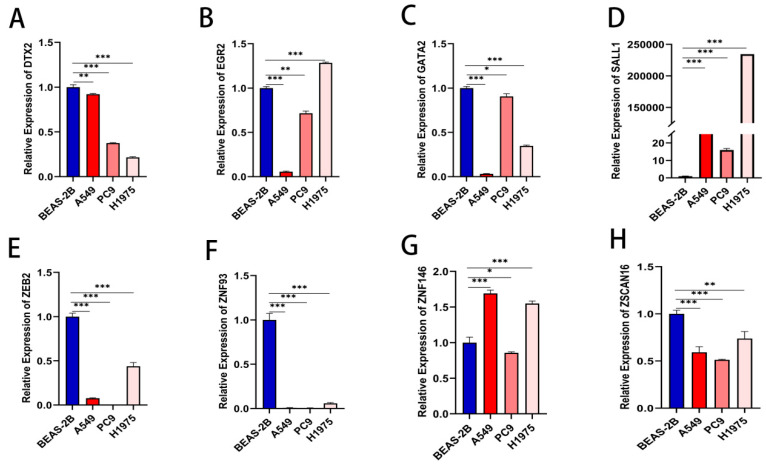
Validation of ZNF gene characteristics in different lung cancer cell lines. DTX2 (**A**), EGR2 (**B**), GATA2 (**C**), SALL1 (**D**), ZEB2 (**E**), ZNF93 (**F**), ZNF146 (**G**), ZSCAN16 (**H**). * *p* < 0.5, ** *p* < 0.01, *** *p* < 0.001.

**Figure 10:**
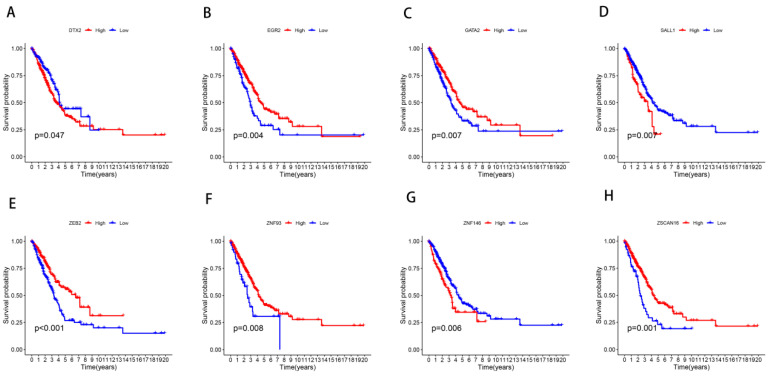
ZNF genes are associated with survival prognosis in LUAD patients. DTX2 (**A**), EGR2 (**B**), GATA2 (**C**), SALL1 (**D**), ZEB2 (**E**), ZNF93 (**F**), ZNF146 (**G**), ZSCAN16 (**H**).

Initial analysis identified eight ZNF genes (|Coef| > 0.1) with tumor-specific expression patterns in TCGA data (Figure 8). Tumor specimens exhibited elevated expression of DTX2, SALL1, ZNF93, ZNF146 and ZSCAN16, contrasting with reduced levels of EGR2, GATA2, and ZEB2. Quantitative PCR validation was performed using normal bronchial epithelium and three LUAD cell lines (A549, H1975, PC9) to confirm model reliability and linical applicability. Figure 9 demonstrates consistent downregulation of GATA2, ZEB2, and SALL1 across malignant cell lines (all *p* < 0.05). EGR2 showed marked suppression in A549/PC9 but not H1975, while ZNF146 displayed elevated expression in A549 and H1975, aligning with TCGA-based predictions. DTX2, ZNF93, and ZSCAN16 exhibited contradictory expression patterns between experimental validation and TCGA data. Metabolic stress deprivation [16] and disrupted mechanical cues [17] likely contribute to transcript abundance variations between clinical specimens and cultured cells. Individual gene scores significantly influenced clinical outcomes in survival analysis (Figure 10).

## 4. Discussion

Previous studies emphasize the oncogenic potential of zinc finger proteins—the most abundant transcriptional regulators in humans—through dysregulated expression patterns [18]. These mechanisms are well-characterized in breast [19], bladder [20], colorectal [21], and prostate malignancies [22]. The functional relevance of ZNFs in lung adenocarcinoma pathogenesis remains insufficiently explored.

Despite therapeutic advancements, lung cancer maintains a dismal prognosis with median overall survival below 5 years [23]. Prognostic stratification systems are essential for personalized therapy to improve clinical outcomes [19]. This model serves as a standalone prognostic indicator, accurately stratifying patients into distinct risk categories with validated predictive capacity. External validation through the GSE68465 cohort corroborated these findings. Collectively, we developed a clinically applicable nomogram for survival prediction and precision therapeutic guidance.

This investigation identified 21 zinc finger protein (ZNF) genes through systematic screening. Eight genes (DTX2, EGR2, GATA2, SALL1, ZEB2, ZNF93, ZNF146, ZSCAN16) demonstrated absolute coefficient values exceeding the 0.1 threshold.

DTX2, an E3 ubiquitin ligase, exhibits significantly elevated expression in non-small-cell lung cancer (NSCLC) and regulates lung cancer cell proliferation through the ferritinophagy and ferroptosis pathways mediated by NCOA4 [24]. Moreover, this gene regulates DNA damage repair [25], consistent with our KEGG enrichment pathway analysis. Our in vitro cellular assays failed to detect upregulated DTX2 levels, potentially reflecting the intricate nature of tumor microenvironmental conditions versus the controlled uniformity of monoculture systems [26]. The early growth response protein 2 (EGR2), a zinc finger-containing transcriptional regulator, serves as a primary regulatory substrate for microRNA-20a, and overexpression of miR-20a leads to a decrease in EGR2 protein levels; moreover, miR-20a exhibits elevated expression in NSCLC [27]. Evidence suggests EGR2 regulates phagocytosis in alveolar macrophages [28], associates STAT6 activation with late-stage macrophage polarization [29], while also playing a critical role in immature T cell differentiation [30].

Regarding the relationship between mRNA and protein levels, it has been documented that an increase in mRNA levels is associated with a sharp rise in protein content, and conversely, a decrease in mRNA levels correlates with reduced protein content [31]. The zinc finger-containing transcriptional regulator GATA2 serves as a pivotal mediator of hematopoiesis regulation and cellular homeostasis, has been shown in multiple studies to exhibit significantly reduced expression in lung cancer [32,33,34]. This observation is highly consistent with our in vitro qPCR results. The GATA2 transcription factor performs diverse functions, participating in hypoxia-induced pulmonary vascular remodeling [35]. More importantly, inhibition of GATA2 markedly inhibits the growth of KRAS-driven lung adenocarcinoma [36]. The chromatin-modulating protein SALL1 exhibits pro-tumorigenic activity in pulmonary carcinoma subtypes classified as non-small cell [37]. However, research regarding this gene in lung cancer or pulmonary contexts remains limited. The majority of literature indicates its critical function within renal tissue [38,39,40,41]. Additionally, SALL1 has been reported to suppress breast cancer progression [42]. The transcriptional regulator ZEB2 harbors a homeodomain motif flanked by two discrete zinc finger structural domains [43]. Notably, several miRNAs suppress migration, invasion, and epithelial-mesenchymal transition in non-small-cell lung cancer through targeting ZEB2 [43,44]. Laboratory-based investigations revealed markedly decreased ZEB2 levels across three LUAD cell models relative to healthy bronchial epithelium. KM estimator plots revealed that ZEB2-driven transcriptional regulation negatively impacts pulmonary adenocarcinoma patient survival trajectories, corroborating existing evidence on its oncogenic regulatory networks [45]. It has been indicated that there is a robust correlation between the ZNF93 gene and the susceptibility to lung cancer [46]; however, the precise mechanisms by which it exerts its influence during the initiation and progression of lung cancer are not yet fully understood. ZNF146 activates the ATR/Chk1 pathway through its overexpression, thereby promoting the occurrence and progression of LUAD. This mechanism has been discovered by researchers Feng Jiang et al. [47]. Beyond its involvement in lung cancer, it accelerates tumor progression across various malignancies, including ovarian cancer [48], hepatocellular carcinoma [49], gastric cancer [50], and colorectal cancer [51]. The ZSCAN16 gene is a subgroup of zinc-finger transcription factors, belonging to the family of DNA-binding transcription factors [52]. Its role has been elucidated in hepatocellular carcinoma [53], ovarian cancer [54], bladder cancer [52], and oral squamous cell carcinoma [55], but its mechanism of action and relative expression levels in lung cancer have not yet been investigated. Prior evidence highlights zinc finger proteins’ (ZNFs) dual regulatory roles in LUAD tumorigenesis and metastatic evolution. This underscores the critical need for deeper exploration of ZNF-based prognostic stratification in pulmonary adenocarcinoma management.

Subsequently, we investigated the potential functions and pathway involvement of ZNFs in LUAD. The GO enrichment analysis results indicate that ZNFs play a significant role in antimicrobial humoral response, humoral immune response, defense response to bacterium, and cilium movement. Differential pathways shared in GSEA and GSVA between high- and low-risk groups include CELL CYCLE, DNA REPLICATION, PYRIMIDINE METABOLISM, DRUG METABOLISM OTHER ENZYMES, and PROTEASOME. Multiple studies have highlighted that intervention in the cell cycle is an effective therapeutic strategy for lung adenocarcinoma, capable of inhibiting cellular proliferation and tumor progression [56,57,58,59]. Pyrimidines are metabolic byproducts of nucleotides and constitute essential structural components of a wide array of key molecules involved in various cellular functions [60]. Reports have indicated that the prognosis of patients with lung adenocarcinoma is correlated with pyrimidine metabolism, and high expression of rate-limiting enzymes in pyrimidine metabolism is a significant factor for poor prognosis in these patients [61]. Furthermore, mutations in the TP53 tumor suppressor gene are considered to be significant factors in tumor progression and survival prognosis for patients with lung adenocarcinoma [62,63]. The TP53 pathway was enriched in the high-risk group, implying that ZNF genes may be involved in the TP53 pathway. However, further evaluation of the roles of these pathways in LUAD is warranted to improve patient OS.

In recent years, infiltrating immune cells in tumors have garnered extensive attention and research, with certain immune cell types being associated with treatment outcomes and OS in cancer patients [64]. Within heterogeneous tissue milieus, quiescent M0 macrophages undergo phenotypic polarization toward M1 (pro-inflammatory) or M2 (anti-inflammatory) subsets, exerting divergent functions across varying pathophysiological contexts [65] and consistently correlating with adverse clinical outcomes [66], while M1 macrophages participate in immune activities by secreting various pro-inflammatory cytokines [67]. The generation of memory B cells is a key characteristic of the adaptive immune response [68]. Memory CD4+ T cells are found throughout the body and play a broad role, participating in inflammatory responses, immune reactions, and promoting the expansion and differentiation of CD8+ T cells [69]. The immune microenvironment exerts pivotal functions in lung adenocarcinoma pathogenesis and advancement, with differential effects on clinical prognosis. Risk stratification analysis demonstrated markedly poorer clinical outcomes in high-risk cohorts compared with low-risk counterparts. Immunophenotyping revealed predominant clustering of M0 and M1 macrophages in high-risk cohorts, whereas low-risk populations demonstrated elevated abundance of memory B lymphocytes and quiescent CD4+ memory T cells.

## 5. Conclusions

This study pioneers a zinc finger protein-based prognostic signature demonstrating robust discriminative capacity and prognostic precision. Clinical implementation of this model enhances therapeutic decision-making and facilitates tailored intervention strategies. Current lung cancer management protocols prioritize early detection, rapid diagnostic confirmation, and prompt therapy initiation. For metastatic or late-stage presentations, conservative strategies including systemic chemo-radiotherapy constitute the primary modality. This work focuses on enhancing screening and diagnostic accuracy, enabling earlier therapeutic intervention while generating prognostic predictions.

Mechanistically, the findings unveil novel therapeutic candidates for lung adenocarcinoma management. Several methodological constraints require acknowledgment. Primarily, the in vitro validation of zinc finger protein-tumorigenesis correlations remains preliminary. While our model (AUC: 0.678–0.717) demonstrated robust performance across External datasets, and the calibration curve shows good prediction accuracy, its construction relied on public genomics cohorts (e.g., TCGA and GEO), introducing inherent limitations: 1. Technical biases: variable RNA-seq protocols may impact gene expression quantification, particularly for low-abundance transcripts like SALL1 and ZNF93. 2. Clinical heterogeneity: inconsistent treatment records and staging criteria limit outcome generalization. 3. Population constraints: Training data primarily represented Western populations, whereas genetic diversity (e.g., Asian cohorts) may alter prognostic weights of key genes. Nevertheless, our model’s prognostic value retains clinical relevance, being derived from real-world cohort data. Additionally, the predictive algorithm derived from retrospective analysis of publicly available cohorts necessitates external validation through prospective multicenter trials. Moreover, within this investigation, functional characterization of the ZNF gene remains incomplete. Consequently, comprehensive investigation into its mechanistic basis warrants further elucidation.

## Data Availability

All analytical datasets employed in this investigation were sourced from open-access repositories. The GSE68465 transcriptomic profile is publicly accessible via the Gene Expression Omnibus repository (Accession: GSE68465; https://www.ncbi.nlm.nih.gov/geo/, accessed on 25 June 2025). LUAD data was collected from The Cancer Genome Atlas (TCGA) database (https://portal.gdc.cancer.gov/).

## Supplementary Materials

The following supporting information can be downloaded at: https://www.mdpi.com/article/10.3390/cancers17132203/s1, Table S1. Symbols and categories of the 1555 ZNFs. Table S2. Primer sequences.
