# Supplementary material for: Zinc Finger Protein-Based Prognostic Signature Predicts Survival in Lung Adenocarcinoma
Source: Cancers (Basel). 2025 Jun 30;17(13):2203. doi: 10.3390/cancers17132203 (PMC12248513; doi:10.3390/cancers17132203)
Supplement: Supplementary file 1 [file cancers-17-02203-s001.zip › cancers-3686843-supplementary.pdf]

Supplementary Table S1. Symbols and categories of the 1555 ZNFs.

HGNC ID	Approved symbol	Approved name
HGNC:1001	BCL6	BCL6 transcription repressor
HGNC:10018	RING1	ring finger protein 1
HGNC:1002	BCL6B	BCL6B transcription repressor
HGNC:10025	RLF	RLF zinc finger
HGNC:10055	RNF10	ring finger protein 10
HGNC:10056	RNF11	ring finger protein 11
HGNC:10057	RNF13	ring finger protein 13
HGNC:10058	RNF14	ring finger protein 14
HGNC:10059	TRIM38	tripartite motif containing 38
HGNC:10060	RNF17	ring finger protein 17
HGNC:10061	RNF2	ring finger protein 2
HGNC:10062	RNF20	ring finger protein 20
HGNC:10063	TRIM34	tripartite motif containing 34
HGNC:10064	TRIM3	tripartite motif containing 3
HGNC:10065	TRIM39	tripartite motif containing 39
HGNC:10066	PCGF3	polycomb group ring finger 3
HGNC:10067	RNF4	ring finger protein 4
HGNC:10068	RNF5	ring finger protein 5
HGNC:10069	RNF6	ring finger protein 6
HGNC:10070	RNF7	ring finger protein 7
HGNC:10071	RNF8	ring finger protein 8
HGNC:10072	TRIM10	tripartite motif containing 10
HGNC:10258	RORA	RAR related orphan receptor A
HGNC:10259	RORB	RAR related orphan receptor B
HGNC:10260	RORC	RAR related orphan receptor C
HGNC:10449	RREB1	ras responsive element binding protein 1
HGNC:10477	RXRA	retinoid X receptor alpha
HGNC:10478	RXRB	retinoid X receptor beta
HGNC:10479	RXRG	retinoid X receptor gamma
HGNC:10480	RYBP	RING1 and YY1 binding protein
HGNC:10524	SALL1	spalt like transcription factor 1
HGNC:10526	SALL2	spalt like transcription factor 2
HGNC:10527	SALL3	spalt like transcription factor 3
HGNC:10573	SCEL	sciellin
HGNC:1066	BMI1	BMI1 proto-oncogene, polycomb ring finger
HGNC:10669	TSHZ1	teashirt zinc finger homeobox 1
HGNC:10784	SCAF11	SR-related CTD associated factor 11
HGNC:10789	SRSF7	serine and arginine rich splicing factor 7
HGNC:1081	BNC1	basonuclin 1
HGNC:10857	SIAH1	siah E3 ubiquitin protein ligase 1
HGNC:10858	SIAH2	siah E3 ubiquitin protein ligase 2
HGNC:1099	BRAP	BRCA1 associated protein

HGNC:1100	BRCA1	BRCA1 DNA repair associated
HGNC:1102	BRD1	bromodomain containing 1
HGNC:1107	ZFP36L1	ZFP36 ring finger protein like 1
HGNC:1108	ZFP36L2	ZFP36 ring finger protein like 2
HGNC:11094	SNAI2	snail family transcriptional repressor 2
HGNC:11099	HLTF	helicase like transcription factor
HGNC:11114	KDM5C	lysine demethylase 5C
HGNC:11115	KDM5D	lysine demethylase 5D
HGNC:11128	SNAI1	snail family transcriptional repressor 1
HGNC:1117	BSN	bassoon presynaptic cytomatrix protein
HGNC:11182	CAPN15	calpain 15
HGNC:11205	SP1	Sp1 transcription factor
HGNC:11206	SP100	SP100 nuclear antigen
HGNC:11207	SP2	Sp2 transcription factor
HGNC:11208	SP3	Sp3 transcription factor
HGNC:11209	SP4	Sp4 transcription factor
HGNC:1123	KLF9	Kruppel like factor 9
HGNC:11280	SQSTM1	sequestosome 1
HGNC:11312	TRIM21	tripartite motif containing 21
HGNC:11547	TAF15	TATA-box binding protein associated factor 15
HGNC:11628	ZNF354A	zinc finger protein 354A
HGNC:11629	TCF19	transcription factor 19
HGNC:11642	ZEB1	zinc finger E-box binding homeobox 1
HGNC:11735	TEX13A	testis expressed 13A
HGNC:11767	TGFB1I1	transforming growth factor beta 1 induced transcript 1
HGNC:1177	ZNHIT2	zinc finger HIT-type containing 2
HGNC:11796	THRA	thyroid hormone receptor alpha
HGNC:11799	THRB	thyroid hormone receptor beta
HGNC:11810	KLF10	Kruppel like factor 10
HGNC:11811	KLF11	Kruppel like factor 11
HGNC:11812	TRIM24	tripartite motif containing 24
HGNC:11955	ZNF384	zinc finger protein 384
HGNC:11992	TOP3A	DNA topoisomerase III alpha
HGNC:12032	TRAF2	TNF receptor associated factor 2
HGNC:12033	TRAF3	TNF receptor associated factor 3
HGNC:12034	TRAF4	TNF receptor associated factor 4
HGNC:12035	TRAF5	TNF receptor associated factor 5
HGNC:12036	TRAF6	TNF receptor associated factor 6
HGNC:12309	ZNHIT3	zinc finger HIT-type containing 3
HGNC:12310	TRIP4	thyroid hormone receptor interactor 4
HGNC:12311	TRIP6	thyroid hormone receptor interactor 6
HGNC:1232	EGLN1	egl-9 family hypoxia inducible factor 1
HGNC:12340	TRPS1	transcriptional repressor GATA binding 1
HGNC:12393	TTC3	tetratricopeptide repeat domain 3
HGNC:12398	TTF2	transcription termination factor 2
HGNC:12556	UHRF1	ubiquitin like with PHD and ring finger domains 1
HGNC:12557	UHRF2	ubiquitin like with PHD and ring finger domains 2
HGNC:12617	USP19	ubiquitin specific peptidase 19

HGNC:12635	UTRN	utrophin
HGNC:12679	VDR	vitamin D receptor
HGNC:12713	VPS41	VPS41 subunit of HOPS complex
HGNC:12766	NSD2	nuclear receptor binding SET domain protein 2
HGNC:12767	NSD3	nuclear receptor binding SET domain protein 3
HGNC:12796	WT1	WT1 transcription factor
HGNC:12856	YY1	YY1 transcription factor
HGNC:12859	RNF103	ring finger protein 103
HGNC:12860	ZBTB14	zinc finger and BTB domain containing 14
HGNC:12861	ZFP3	ZFP3 zinc finger protein
HGNC:12862	ZFP36	ZFP36 ring finger protein
HGNC:12863	ZFP37	ZFP37 zinc finger protein
HGNC:12865	ZFP92	ZFP92 zinc finger protein
HGNC:12866	ZNF235	zinc finger protein 235
HGNC:12867	ZKSCAN5	zinc finger with KRAB and SCAN domains 5
HGNC:12868	ZFPL1	zinc finger protein like 1
HGNC:12869	ZFX	zinc finger protein X-linked
HGNC:12870	ZFY	zinc finger protein Y-linked
HGNC:12871	ZHX1	zinc fingers and homeoboxes 1
HGNC:12872	ZIC1	Zic family member 1
HGNC:12873	ZIC2	Zic family member 2
HGNC:12874	ZIC3	Zic family member 3
HGNC:12875	ZIM2	zinc finger imprinted 2
HGNC:12879	ZNF10	zinc finger protein 10
HGNC:12880	ZNF100	zinc finger protein 100
HGNC:12881	ZNF101	zinc finger protein 101
HGNC:12886	ZNF106	zinc finger protein 106
HGNC:12887	ZNF107	zinc finger protein 107
HGNC:12892	ZNF112	zinc finger protein 112
HGNC:12894	ZNF114	zinc finger protein 114
HGNC:12897	ZNF117	zinc finger protein 117
HGNC:12902	ZNF12	zinc finger protein 12
HGNC:12904	ZNF121	zinc finger protein 121
HGNC:12907	ZNF124	zinc finger protein 124
HGNC:12915	ZNF131	zinc finger protein 131
HGNC:12916	ZNF132	zinc finger protein 132
HGNC:12917	ZNF133	zinc finger protein 133
HGNC:12918	ZNF134	zinc finger protein 134
HGNC:12919	ZNF135	zinc finger protein 135
HGNC:12920	ZNF136	zinc finger protein 136
HGNC:12922	ZNF138	zinc finger protein 138
HGNC:12924	ZNF14	zinc finger protein 14
HGNC:12925	ZNF140	zinc finger protein 140
HGNC:12926	ZNF141	zinc finger protein 141
HGNC:12927	ZNF142	zinc finger protein 142
HGNC:12928	ZNF143	zinc finger protein 143
HGNC:12929	PCGF2	polycomb group ring finger 2
HGNC:12930	ZBTB16	zinc finger and BTB domain containing 16

HGNC:12931	ZNF146	zinc finger protein 146
HGNC:12932	TRIM25	tripartite motif containing 25
HGNC:12933	ZNF148	zinc finger protein 148
HGNC:12936	ZBTB17	zinc finger and BTB domain containing 17
HGNC:12939	ZNF154	zinc finger protein 154
HGNC:12940	ZNF155	zinc finger protein 155
HGNC:12942	ZNF157	zinc finger protein 157
HGNC:12945	ZNF708	zinc finger protein 708
HGNC:12947	ZNF16	zinc finger protein 16
HGNC:12948	ZNF160	zinc finger protein 160
HGNC:12949	VEZF1	vascular endothelial zinc finger 1
HGNC:12950	SF1	splicing factor 1
HGNC:12953	ZNF165	zinc finger protein 165
HGNC:12955	ZKSCAN7	zinc finger with KRAB and SCAN domains 7
HGNC:12957	ZNF169	zinc finger protein 169
HGNC:12958	ZNF17	zinc finger protein 17
HGNC:12962	TRIM26	tripartite motif containing 26
HGNC:12963	ZNF174	zinc finger protein 174
HGNC:12964	ZNF175	zinc finger protein 175
HGNC:12966	ZNF177	zinc finger protein 177
HGNC:12968	RNF112	ring finger protein 112
HGNC:12969	ZNF18	zinc finger protein 18
HGNC:12970	ZNF180	zinc finger protein 180
HGNC:12971	ZNF181	zinc finger protein 181
HGNC:12974	RNF113A	ring finger protein 113A
HGNC:12975	ZNF184	zinc finger protein 184
HGNC:12976	ZNF185	zinc finger protein 185 with LIM domain
HGNC:12978	ZSCAN26	zinc finger and SCAN domain containing 26
HGNC:12980	ZNF189	zinc finger protein 189
HGNC:12981	ZNF19	zinc finger protein 19
HGNC:12983	ZKSCAN8	zinc finger with KRAB and SCAN domains 8
HGNC:12984	ZSCAN9	zinc finger and SCAN domain containing 9
HGNC:12986	ZNF195	zinc finger protein 195
HGNC:12988	ZNF197	zinc finger protein 197
HGNC:12989	ZMYM2	zinc finger MYM-type containing 2
HGNC:12991	ZNF2	zinc finger protein 2
HGNC:12992	ZNF20	zinc finger protein 20
HGNC:12993	ZNF200	zinc finger protein 200
HGNC:12994	ZNF202	zinc finger protein 202
HGNC:12996	ZNF205	zinc finger protein 205
HGNC:12997	ZSCAN10	zinc finger and SCAN domain containing 10
HGNC:12998	ZNF207	zinc finger protein 207
HGNC:12999	ZNF208	zinc finger protein 208
HGNC:13001	ZNF182	zinc finger protein 182
HGNC:13003	ZNF211	zinc finger protein 211
HGNC:13004	ZNF212	zinc finger protein 212
HGNC:13005	ZNF213	zinc finger protein 213
HGNC:13006	ZNF214	zinc finger protein 214

HGNC:13007	ZNF215	zinc finger protein 215
HGNC:13008	ZFAND5	zinc finger AN1-type containing 5
HGNC:13009	ZNF217	zinc finger protein 217
HGNC:13010	TSHZ2	teashirt zinc finger homeobox 2
HGNC:13011	ZNF219	zinc finger protein 219
HGNC:13012	ZNF22	zinc finger protein 22
HGNC:13013	KAT6A	lysine acetyltransferase 6A
HGNC:13014	ZNF221	zinc finger protein 221
HGNC:13015	ZNF222	zinc finger protein 222
HGNC:13016	ZNF223	zinc finger protein 223
HGNC:13017	ZNF224	zinc finger protein 224
HGNC:13018	ZNF225	zinc finger protein 225
HGNC:13019	ZNF226	zinc finger protein 226
HGNC:13020	ZNF227	zinc finger protein 227
HGNC:13022	ZNF229	zinc finger protein 229
HGNC:13023	ZNF23	zinc finger protein 23
HGNC:13024	ZNF230	zinc finger protein 230
HGNC:13026	ZNF232	zinc finger protein 232
HGNC:13027	ZNF234	zinc finger protein 234
HGNC:13028	ZNF236	zinc finger protein 236
HGNC:13029	ZMYM5	zinc finger MYM-type containing 5
HGNC:13030	ZBTB18	zinc finger and BTB domain containing 18
HGNC:13031	ZNF239	zinc finger protein 239
HGNC:13032	ZNF24	zinc finger protein 24
HGNC:13041	ZNF248	zinc finger protein 248
HGNC:13043	ZNF25	zinc finger protein 25
HGNC:13044	ZNF250	zinc finger protein 250
HGNC:13045	ZNF251	zinc finger protein 251
HGNC:13047	ZNF254	zinc finger protein 254
HGNC:13049	ZNF256	zinc finger protein 256
HGNC:13050	ZMYM6	zinc finger MYM-type containing 6
HGNC:13051	ZPR1	ZPR1 zinc finger
HGNC:13053	ZNF26	zinc finger protein 26
HGNC:13054	ZMYM3	zinc finger MYM-type containing 3
HGNC:13055	ZMYM4	zinc finger MYM-type containing 4
HGNC:13056	ZNF263	zinc finger protein 263
HGNC:13057	ZNF264	zinc finger protein 264
HGNC:13058	ZRANB2	zinc finger RANBP2-type containing 2
HGNC:13059	ZNF266	zinc finger protein 266
HGNC:13060	ZNF267	zinc finger protein 267
HGNC:13061	ZNF268	zinc finger protein 268
HGNC:13067	ZNF273	zinc finger protein 273
HGNC:13068	ZNF274	zinc finger protein 274
HGNC:13069	ZNF275	zinc finger protein 275
HGNC:13070	ZNF277	zinc finger protein 277
HGNC:13071	PATZ1	POZ/BTB and AT hook containing zinc finger 1
HGNC:13073	ZNF28	zinc finger protein 28
HGNC:13075	ZNF281	zinc finger protein 281

HGNC:13076	ZNF282	zinc finger protein 282
HGNC:13077	ZNF283	zinc finger protein 283
HGNC:13078	ZNF284	zinc finger protein 284
HGNC:13079	ZNF285	zinc finger protein 285
HGNC:13081	SCAPER	S-phase cyclin A associated protein in the ER
HGNC:13082	LTN1	listerin E3 ubiquitin protein ligase 1
HGNC:13083	ZBTB21	zinc finger and BTB domain containing 21
HGNC:13085	ZBTB22	zinc finger and BTB domain containing 22
HGNC:13089	ZNF3	zinc finger protein 3
HGNC:13090	ZNF30	zinc finger protein 30
HGNC:13091	ZNF300	zinc finger protein 300
HGNC:13093	ZSCAN20	zinc finger and SCAN domain containing 20
HGNC:13094	RNF114	ring finger protein 114
HGNC:13095	ZNF32	zinc finger protein 32
HGNC:13096	ZNF33A	zinc finger protein 33A
HGNC:13097	ZNF33B	zinc finger protein 33B
HGNC:13098	ZNF34	zinc finger protein 34
HGNC:13099	ZNF35	zinc finger protein 35
HGNC:13101	ZKSCAN1	zinc finger with KRAB and SCAN domains 1
HGNC:13102	ZNF37A	zinc finger protein 37A
HGNC:13104	ZSCAN21	zinc finger and SCAN domain containing 21
HGNC:13107	ZNF41	zinc finger protein 41
HGNC:13108	MZF1	myeloid zinc finger 1
HGNC:13109	ZNF43	zinc finger protein 43
HGNC:13110	ZNF44	zinc finger protein 44
HGNC:13111	ZNF45	zinc finger protein 45
HGNC:13112	ZBTB25	zinc finger and BTB domain containing 25
HGNC:13114	ZNF48	zinc finger protein 48
HGNC:13125	ZNF57	zinc finger protein 57
HGNC:13128	ZNF711	zinc finger protein 711
HGNC:13135	ZNF66	zinc finger protein 66
HGNC:13138	ZNF69	zinc finger protein 69
HGNC:13139	ZNF7	zinc finger protein 7
HGNC:13140	ZNF70	zinc finger protein 70
HGNC:13141	ZNF71	zinc finger protein 71
HGNC:13144	ZNF74	zinc finger protein 74
HGNC:13145	ZNF75D	zinc finger protein 75D
HGNC:13146	ZNF75A	zinc finger protein 75a
HGNC:13149	ZNF76	zinc finger protein 76
HGNC:13150	ZNF77	zinc finger protein 77
HGNC:13153	ZNF79	zinc finger protein 79
HGNC:13154	ZNF8	zinc finger protein 8
HGNC:13155	ZNF80	zinc finger protein 80
HGNC:13156	ZNF81	zinc finger protein 81
HGNC:13158	ZNF83	zinc finger protein 83
HGNC:13159	ZNF84	zinc finger protein 84
HGNC:13160	ZNF85	zinc finger protein 85
HGNC:13164	CNBP	CCHC-type zinc finger nucleic acid binding protein

HGNC:13165	ZNF90	zinc finger protein 90
HGNC:13166	ZNF91	zinc finger protein 91
HGNC:13168	ZNF92	zinc finger protein 92
HGNC:13169	ZNF93	zinc finger protein 93
HGNC:13172	ZSCAN12	zinc finger and SCAN domain containing 12
HGNC:13174	ZNF98	zinc finger protein 98
HGNC:13175	ZNF99	zinc finger protein 99
HGNC:13176	IKZF1	IKAROS family zinc finger 1
HGNC:13177	IKZF2	IKAROS family zinc finger 2
HGNC:13178	IKZF3	IKAROS family zinc finger 3
HGNC:13179	IKZF4	IKAROS family zinc finger 4
HGNC:13180	ZFYVE1	zinc finger FYVE-type containing 1
HGNC:13198	ZXDA	zinc finger X-linked duplicated A
HGNC:13199	ZXDB	zinc finger X-linked duplicated B
HGNC:13200	ZYX	zyxin
HGNC:13221	BCL11A	BAF chromatin remodeling complex subunit BCL11A
HGNC:13222	BCL11B	BAF chromatin remodeling complex subunit BCL11B
HGNC:13324	INO80B	INO80 complex subunit B
HGNC:13406	PCLO	piccolo presynaptic cytomatrix protein
HGNC:13429	RLIM	ring finger protein, LIM domain interacting
HGNC:13430	TRIM17	tripartite motif containing 17
HGNC:13431	TRIM49	tripartite motif containing 49
HGNC:13432	RNF19A	ring finger protein 19A, RBR E3 ubiquitin protein ligase
HGNC:13497	ZNF253	zinc finger protein 253
HGNC:13498	ZNF257	zinc finger protein 257
HGNC:13499	ZNF260	zinc finger protein 260
HGNC:13501	ZNF286A	zinc finger protein 286A
HGNC:13502	ZNF287	zinc finger protein 287
HGNC:13503	ZBTB20	zinc finger and BTB domain containing 20
HGNC:13505	ZNF304	zinc finger protein 304
HGNC:13506	FEZF2	FEZ family zinc finger 2
HGNC:13507	ZNF317	zinc finger protein 317
HGNC:13561	HIVEP3	HIVEP zinc finger 3
HGNC:13578	ZNF318	zinc finger protein 318
HGNC:13606	KDM2A	lysine demethylase 2A
HGNC:13610	KDM2B	lysine demethylase 2B
HGNC:13644	ZNF319	zinc finger protein 319
HGNC:13672	KLF13	Kruppel like factor 13
HGNC:13702	BIRC7	baculoviral IAP repeat containing 7
HGNC:13723	CTCF	CCCTC-binding factor
HGNC:13726	KMT2C	lysine methyltransferase 2C
HGNC:13779	RNF24	ring finger protein 24
HGNC:13842	ZNF320	zinc finger protein 320
HGNC:13843	ZNF316	zinc finger protein 316
HGNC:13847	ZNF311	zinc finger protein 311
HGNC:13848	ZNF302	zinc finger protein 302
HGNC:13851	ZBED9	zinc finger BED-type containing 9
HGNC:13853	ZKSCAN3	zinc finger with KRAB and SCAN domains 3

HGNC:13854	ZKSCAN4	zinc finger with KRAB and SCAN domains 4
HGNC:13992	PDLIM2	PDZ and LIM domain 2
HGNC:13994	PRDM9	PR/SET domain 9
HGNC:13995	PRDM10	PR/SET domain 10
HGNC:13997	PRDM12	PR/SET domain 12
HGNC:13999	PRDM15	PR/SET domain 15
HGNC:14000	PRDM16	PR/SET domain 16
HGNC:14001	PRDM14	PR/SET domain 14
HGNC:14061	LPXN	leupaxin
HGNC:14096	ZNF324	zinc finger protein 324
HGNC:14097	ZSCAN31	zinc finger and SCAN domain containing 31
HGNC:14104	ZNF326	zinc finger protein 326
HGNC:14184	UNKL	unk like zinc finger
HGNC:14186	OVOL3	ovo like zinc finger 3
HGNC:14209	ZNF329	zinc finger protein 329
HGNC:14215	TRIM55	tripartite motif containing 55
HGNC:14216	LHX5	LIM homeobox 5
HGNC:14222	LHX9	LIM homeobox 9
HGNC:14234	NSD1	nuclear receptor binding SET domain protein 1
HGNC:14255	BRPF1	bromodomain and PHD finger containing 1
HGNC:14256	BRPF3	bromodomain and PHD finger containing 3
HGNC:14283	IKZF5	IKAROS family zinc finger 5
HGNC:14303	XIRP2	xin actin binding repeat containing 2
HGNC:14452	ANAPC11	anaphase promoting complex subunit 11
HGNC:14529	SP5	Sp5 transcription factor
HGNC:14530	SP6	Sp6 transcription factor
HGNC:14536	KLF15	Kruppel like factor 15
HGNC:14539	RNF213	ring finger protein 213
HGNC:14583	VPS11	VPS11 core subunit of CORVET and HOPS complexes
HGNC:14587	ING3	inhibitor of growth family member 3
HGNC:14620	TES	testin LIM domain protein
HGNC:14646	RNF26	ring finger protein 26
HGNC:14662	RNF25	ring finger protein 25
HGNC:14663	TRIM64	tripartite motif containing 64
HGNC:14673	FYCO1	FYVE and coiled-coil domain autophagy adaptor 1
HGNC:14677	DEAF1	DEAF1 transcription factor
HGNC:14878	BIRC8	baculoviral IAP repeat containing 8
HGNC:14881	ZEB2	zinc finger E-box binding homeobox 2
HGNC:14955	MYNN	myoneurin
HGNC:14983	ZFP91	ZFP91 zinc finger protein, atypical E3 ubiquitin ligase
HGNC:1535	RUNX1T1	RUNX1 partner transcriptional co-repressor 1
HGNC:1536	CBFA2T2	CBFA2/RUNX1 partner transcriptional co-repressor 2
HGNC:1537	CBFA2T3	CBFA2/RUNX1 partner transcriptional co-repressor 3
HGNC:1541	CBL	Cbl proto-oncogene
HGNC:1542	CBLB	Cbl proto-oncogene B
HGNC:15462	ZNF330	zinc finger protein 330
HGNC:15489	ZNF331	zinc finger protein 331
HGNC:15513	SMYD3	SET and MYND domain containing 3

HGNC:15528	CGRRF1	cell growth regulator with ring finger domain 1
HGNC:15572	RTP3	receptor transporter protein 3
HGNC:15579	TRIM8	tripartite motif containing 8
HGNC:15624	ZNF333	zinc finger protein 333
HGNC:15710	LDB3	LIM domain binding 3
HGNC:15766	ADNP	activity dependent neuroprotector homeobox
HGNC:15802	GATA5	GATA binding protein 5
HGNC:15804	OVOL2	ovo like zinc finger 2
HGNC:15806	ZNF334	zinc finger protein 334
HGNC:15807	ZNF335	zinc finger protein 335
HGNC:15808	GZF1	GDNF inducible zinc finger protein 1
HGNC:15809	ZNF337	zinc finger protein 337
HGNC:15830	OSR2	odd-skipped related transciption factor 2
HGNC:15840	KMT2B	lysine methyltransferase 2B
HGNC:15864	RBCK1	RANBP2-type and C3HC4-type zinc finger containing 1
HGNC:15905	L3MBTL1	L3MBTL histone methyl-lysine binding protein 1
HGNC:15924	SALL4	spalt like transcription factor 4
HGNC:15935	ZHX3	zinc fingers and homeoboxes 3
HGNC:15940	ZFP64	ZFP64 zinc finger protein
HGNC:15948	ZGPAT	zinc finger CCCH-type and G-patch domain containing
HGNC:15950	SCRT1	scratch family transcriptional repressor 1
HGNC:15952	SCRT2	scratch family transcriptional repressor 2
HGNC:15961	CBLC	Cbl proto-oncogene C
HGNC:15973	DTX2	deltex E3 ubiquitin ligase 2
HGNC:15974	TRIM2	tripartite motif containing 2
HGNC:15981	ZNF296	zinc finger protein 296
HGNC:15986	LIN28A	lin-28 homolog A
HGNC:15992	ZNF341	zinc finger protein 341
HGNC:16007	TRIM63	tripartite motif containing 63
HGNC:16008	TRIM54	tripartite motif containing 54
HGNC:16017	ZNF343	zinc finger protein 343
HGNC:16027	FGD3	FYVE, RhoGEF and PH domain containing 3
HGNC:16031	RNF31	ring finger protein 31
HGNC:16052	ZNF444	zinc finger protein 444
HGNC:16063	MLLT10	MLLT10 histone lysine methyltransferase DOT1L cofactor
HGNC:16084	LIMS2	LIM zinc finger domain containing 2
HGNC:16094	ZBTB46	zinc finger and BTB domain containing 46
HGNC:16098	PHF20	PHD finger protein 20
HGNC:16155	ZSWIM1	zinc finger SWIM-type containing 1
HGNC:16157	ZSWIM3	zinc finger SWIM-type containing 3
HGNC:16167	ZNF831	zinc finger protein 831
HGNC:16230	ZCCHC3	zinc finger CCHC-type containing 3
HGNC:16234	CTCFL	CCCTC-binding factor like
HGNC:16258	SMYD5	SMYD family member 5
HGNC:16275	TRIM4	tripartite motif containing 4
HGNC:16276	TRIM5	tripartite motif containing 5
HGNC:16277	TRIM6	tripartite motif containing 6
HGNC:16278	TRIM7	tripartite motif containing 7

HGNC:16280	TRIM36	tripartite motif containing 36
HGNC:16281	TRIM11	tripartite motif containing 11
HGNC:16284	TRIM15	tripartite motif containing 15
HGNC:16285	TRIM35	tripartite motif containing 35
HGNC:16288	TRIM9	tripartite motif containing 9
HGNC:16289	TRIM31	tripartite motif containing 31
HGNC:16290	TRIM33	tripartite motif containing 33
HGNC:16366	ZIM3	zinc finger imprinted 3
HGNC:16367	ZNF345	zinc finger protein 345
HGNC:16379	TRIM22	tripartite motif containing 22
HGNC:16380	TRIM32	tripartite motif containing 32
HGNC:16384	TRIM28	tripartite motif containing 28
HGNC:16403	ZNF346	zinc finger protein 346
HGNC:16447	ZNF347	zinc finger protein 347
HGNC:16449	RFPL4A	ret finger protein like 4A
HGNC:16493	ZMIZ1	zinc finger MIZ-type containing 1
HGNC:16501	PDLIM4	PDZ and LIM domain 4
HGNC:16516	KLF3	Kruppel like factor 3
HGNC:16648	PJA1	praja ring finger ubiquitin ligase 1
HGNC:16656	ZNF350	zinc finger protein 350
HGNC:16682	ZBTB33	zinc finger and BTB domain containing 33
HGNC:16700	ZFPM2	zinc finger protein, FOG family member 2
HGNC:16734	MEX3D	mex-3 RNA binding family member D
HGNC:16736	ZNF354C	zinc finger protein 354C
HGNC:16740	ZBTB11	zinc finger and BTB domain containing 11
HGNC:16744	CIZ1	CDKN1A interacting zinc finger protein 1
HGNC:16746	MBNL2	muscleblind like splicing regulator 2
HGNC:16747	ZNF589	zinc finger protein 589
HGNC:16759	ZNF544	zinc finger protein 544
HGNC:16762	ZNF423	zinc finger protein 423
HGNC:16763	ZBTB32	zinc finger and BTB domain containing 32
HGNC:16764	ZBTB6	zinc finger and BTB domain containing 6
HGNC:16805	PRICKLE4	prickle planar cell polarity protein 4
HGNC:16816	CHD5	chromodomain helicase DNA binding protein 5
HGNC:16838	ZNF358	zinc finger protein 358
HGNC:16857	KLF16	Kruppel like factor 16
HGNC:16861	PIAS3	protein inhibitor of activated STAT 3
HGNC:16867	RNF40	ring finger protein 40
HGNC:16878	HELZ	helicase with zinc finger
HGNC:16932	NEBL	nebulette
HGNC:16966	ZMYND11	zinc finger MYND-type containing 11
HGNC:17002	PIAS4	protein inhibitor of activated STAT 4
HGNC:17016	KAT7	lysine acetyltransferase 7
HGNC:17019	PRICKLE1	prickle planar cell polarity protein 1
HGNC:17023	RNF139	ring finger protein 139
HGNC:17024	PHF11	PHD finger protein 11
HGNC:17075	TAB2	TGF-beta activated kinase 1 (MAP3K7) binding protein 2
HGNC:17101	SUZ12	SUZ12 polycomb repressive complex 2 subunit

HGNC:17118	RNF32	ring finger protein 32
HGNC:17133	SP140	SP140 nuclear body protein
HGNC:17197	ZNF354B	zinc finger protein 354B
HGNC:17267	RNF113B	ring finger protein 113B
HGNC:17277	ZFR	zinc finger RNA binding protein
HGNC:17297	RNF34	ring finger protein 34
HGNC:17303	TAF3	TATA-box binding protein associated factor 3
HGNC:17311	PIAS2	protein inhibitor of activated STAT 2
HGNC:17321	SP7	Sp7 transcription factor
HGNC:17363	YAF2	YY1 associated factor 2
HGNC:17364	DBF4	DBF4 zinc finger
HGNC:17371	FHL5	four and a half LIM domains 5
HGNC:17384	RNF111	ring finger protein 111
HGNC:17393	MARCHF7	membrane associated ring-CH-type finger 7
HGNC:17407	ZC3H12B	zinc finger CCCH-type containing 12B
HGNC:17409	ZNF382	zinc finger protein 382
HGNC:17427	DPF3	double PHD fingers 3
HGNC:17440	COP1	COP1 E3 ubiquitin ligase
HGNC:17453	TRIM74	tripartite motif containing 74
HGNC:17468	PDLIM5	PDZ and LIM domain 5
HGNC:17479	RCHY1	ring finger and CHY zinc finger domain containing 1
HGNC:17481	PJA2	praja ring finger ubiquitin ligase 2
HGNC:17521	ZNF385A	zinc finger protein 385A
HGNC:17539	INSM2	INSM transcriptional repressor 2
HGNC:17582	KAT6B	lysine acetyltransferase 6B
HGNC:17613	BFAR	bifunctional apoptosis regulator
HGNC:17615	PCGF1	polycomb group ring finger 1
HGNC:17625	ZNF674	zinc finger protein 674
HGNC:17650	SH3RF1	SH3 domain containing ring finger 1
HGNC:17680	RBAK	RB associated KRAB zinc finger
HGNC:17704	PDZRN3	PDZ domain containing ring finger 3
HGNC:17726	ZNRF4	zinc and ring finger 4
HGNC:17751	CRIP3	cysteine rich protein 3
HGNC:17765	RNF138	ring finger protein 138
HGNC:17777	UBOX5	U-box domain containing 5
HGNC:17801	ZFP28	ZFP28 zinc finger protein
HGNC:17806	CYHR1	cysteine and histidine rich 1
HGNC:17808	ZC3H4	zinc finger CCCH-type containing 4
HGNC:17850	HINFP	histone H4 transcription factor
HGNC:17857	TRIM69	tripartite motif containing 69
HGNC:17883	DBF4B	DBF4 zinc finger B
HGNC:17889	APEX2	apurinic/apyrimidinic endodeoxyribonuclease 2
HGNC:17894	ZNF638	zinc finger protein 638
HGNC:17908	ZBTB43	zinc finger and BTB domain containing 43
HGNC:17916	ZDHHC1	zinc finger DHHC-type containing 1
HGNC:17922	REPIN1	replication initiator 1
HGNC:17933	KAT8	lysine acetyltransferase 8
HGNC:18000	PHF5A	PHD finger protein 5A

HGNC:18019	ZFAND3	zinc finger AN1-type containing 3
HGNC:18039	KDM5B	lysine demethylase 5B
HGNC:18052	RNF38	ring finger protein 38
HGNC:18064	RNF39	ring finger protein 39
HGNC:18078	ZBTB7A	zinc finger and BTB domain containing 7A
HGNC:18079	ZNF362	zinc finger protein 362
HGNC:18118	RSF1	remodeling and spacing factor 1
HGNC:18126	ZNRF3	zinc and ring finger 3
HGNC:18145	PHF6	PHD finger protein 6
HGNC:18154	RNF115	ring finger protein 115
HGNC:18162	TRIM73	tripartite motif containing 73
HGNC:18190	ZNF648	zinc finger protein 648
HGNC:18194	ZNF365	zinc finger protein 365
HGNC:18224	ZRANB1	zinc finger RANBP2-type containing 1
HGNC:18250	PHF10	PHD finger protein 10
HGNC:18261	NPLOC4	NPL4 homolog, ubiquitin recognition factor
HGNC:18278	RAD18	RAD18 E3 ubiquitin protein ligase
HGNC:18280	RNF130	ring finger protein 130
HGNC:18316	ZNF366	zinc finger protein 366
HGNC:18320	ZNF367	zinc finger protein 367
HGNC:18373	ZNF398	zinc finger protein 398
HGNC:18401	RNF41	ring finger protein 41
HGNC:18410	ZNF292	zinc finger protein 292
HGNC:18411	SNAI3	snail family transcriptional repressor 3
HGNC:18412	ZDHHC17	zinc finger DHHC-type palmitoyltransferase 17
HGNC:18413	ZDHHC13	zinc finger DHHC-type palmitoyltransferase 13
HGNC:18452	ZNRF1	zinc and ring finger 1
HGNC:18458	PHF7	PHD finger protein 7
HGNC:18459	ZDHHC7	zinc finger DHHC-type palmitoyltransferase 7
HGNC:18469	ZDHHC2	zinc finger DHHC-type palmitoyltransferase 2
HGNC:18470	ZDHHC3	zinc finger DHHC-type palmitoyltransferase 3
HGNC:18471	ZDHHC4	zinc finger DHHC-type palmitoyltransferase 4
HGNC:18472	ZDHHC5	zinc finger DHHC-type palmitoyltransferase 5
HGNC:18474	ZDHHC8	zinc finger DHHC-type palmitoyltransferase 8
HGNC:18475	ZDHHC9	zinc finger DHHC-type palmitoyltransferase 9
HGNC:18505	RNF43	ring finger protein 43
HGNC:18513	ZHX2	zinc fingers and homeoboxes 2
HGNC:18524	ISL2	ISL LIM homeobox 2
HGNC:18541	KMT2E	lysine methyltransferase 2E (inactive)
HGNC:18595	HIC2	HIC ZBTB transcriptional repressor 2
HGNC:18597	ZNF280A	zinc finger protein 280A
HGNC:18609	ZNF383	zinc finger protein 383
HGNC:18668	ZBTB7B	zinc finger and BTB domain containing 7B
HGNC:18695	ST18	ST18 C2H2C-type zinc finger transcription factor
HGNC:18726	NFXL1	nuclear transcription factor, X-box binding like 1
HGNC:18736	TRIM40	tripartite motif containing 40
HGNC:18737	ZNF395	zinc finger protein 395
HGNC:18779	ZNF391	zinc finger protein 391

HGNC:18791	ZFP57	ZFP57 zinc finger protein
HGNC:18818	ZNF397	zinc finger protein 397
HGNC:18824	ZNF396	zinc finger protein 396
HGNC:18830	KLF17	Kruppel like factor 17
HGNC:18832	ZNF394	zinc finger protein 394
HGNC:19013	TRIM41	tripartite motif containing 41
HGNC:19014	TRIM42	tripartite motif containing 42
HGNC:19015	TRIM43	tripartite motif containing 43
HGNC:19016	TRIM44	tripartite motif containing 44
HGNC:19017	TRIM50	tripartite motif containing 50
HGNC:19018	TRIM45	tripartite motif containing 45
HGNC:19019	TRIM46	tripartite motif containing 46
HGNC:19020	TRIM47	tripartite motif containing 47
HGNC:19021	TRIM48	tripartite motif containing 48
HGNC:19023	TRIM51	tripartite motif-containing 51
HGNC:19024	TRIM52	tripartite motif containing 52
HGNC:19028	TRIM56	tripartite motif containing 56
HGNC:19066	ZBTB12	zinc finger and BTB domain containing 12
HGNC:19088	ASH1L	ASH1 like histone lysine methyltransferase
HGNC:19117	FGD5	FYVE, RhoGEF and PH domain containing 5
HGNC:19125	FGD4	FYVE, RhoGEF and PH domain containing 4
HGNC:19158	ZDHHC11	zinc finger DHHC-type containing 11
HGNC:19159	ZDHHC12	zinc finger DHHC-type palmitoyltransferase 12
HGNC:19160	ZDHHC6	zinc finger DHHC-type palmitoyltransferase 6
HGNC:1918	CHD3	chromodomain helicase DNA binding protein 3
HGNC:19180	RNF44	ring finger protein 44
HGNC:1919	CHD4	chromodomain helicase DNA binding protein 4
HGNC:19195	ABLIM2	actin binding LIM protein family member 2
HGNC:19196	SP8	Sp8 transcription factor
HGNC:19336	SHPRH	SNF2 histone linker PHD RING helicase
HGNC:19412	ZMYND10	zinc finger MYND-type containing 10
HGNC:19417	ZNF404	zinc finger protein 404
HGNC:19421	ING5	inhibitor of growth family member 5
HGNC:19423	ING4	inhibitor of growth family member 4
HGNC:19437	CCNB1IP1	cyclin B1 interacting protein 1
HGNC:19760	RUFY1	RUN and FYVE domain containing 1
HGNC:19761	RUFY2	RUN and FYVE domain containing 2
HGNC:19762	ZFPM1	zinc finger protein, FOG family member 1
HGNC:19883	ZBTB49	zinc finger and BTB domain containing 49
HGNC:19899	ZFAT	zinc finger and AT-hook domain containing
HGNC:19904	ZNF407	zinc finger protein 407
HGNC:20041	ZNF408	zinc finger protein 408
HGNC:20106	ZDHHC22	zinc finger DHHC-type palmitoyltransferase 22
HGNC:20144	ZNF410	zinc finger protein 410
HGNC:20152	ZFHX2	zinc finger homeobox 2
HGNC:20164	HOMEZ	homeobox and leucine zipper encoding
HGNC:20225	DPF1	double PHD fingers 1
HGNC:20250	AJUBA	ajuba LIM protein

HGNC:20254	MGRN1	mahogunin ring finger 1
HGNC:20259	ZBTB1	zinc finger and BTB domain containing 1
HGNC:20308	OBI1	ORC ubiquitin ligase 1
HGNC:20311	CHAMP1	chromosome alignment maintaining phosphoprotein 1
HGNC:20322	ZIC5	Zic family member 5
HGNC:20327	RBM26	RNA binding motif protein 26
HGNC:20338	G2E3	G2/M-phase specific E3 ubiquitin protein ligase
HGNC:20340	PRICKLE2	prickle planar cell polarity protein 2
HGNC:20341	ZDHHC14	zinc finger DHHC-type palmitoyltransferase 14
HGNC:20342	ZDHHC15	zinc finger DHHC-type palmitoyltransferase 15
HGNC:20345	ZNF839	zinc finger protein 839
HGNC:20354	ZC2HC1C	zinc finger C2HC-type containing 1C
HGNC:20368	ZC3H13	zinc finger CCCH-type containing 13
HGNC:20393	ZIC4	Zic family member 4
HGNC:20421	LNX2	ligand of numb-protein X 2
HGNC:20429	ZNF676	zinc finger protein 676
HGNC:20436	ZAR1	zygote arrest 1
HGNC:20438	RNF212B	ring finger protein 212B
HGNC:20451	WDFY1	WD repeat and FYVE domain containing 1
HGNC:20455	CHFR	checkpoint with forkhead and ring finger domains
HGNC:20456	TRAF7	TNF receptor associated factor 7
HGNC:20457	RNF144A	ring finger protein 144A
HGNC:20482	WDFY2	WD repeat and FYVE domain containing 2
HGNC:20509	ZC3H14	zinc finger CCCH-type containing 14
HGNC:20564	MBNL3	muscleblind like splicing regulator 3
HGNC:20589	KCMF1	potassium channel modulatory factor 1
HGNC:20619	MICAL1	microtubule associated monooxygenase, calponin and LIM domain containing 1
HGNC:20629	ZNF709	zinc finger protein 709
HGNC:20630	ZNF414	zinc finger protein 414
HGNC:20636	ZNF415	zinc finger protein 415
HGNC:20645	ZNF416	zinc finger protein 416
HGNC:20646	ZNF417	zinc finger protein 417
HGNC:20647	ZNF418	zinc finger protein 418
HGNC:20648	ZNF419	zinc finger protein 419
HGNC:20649	ZNF420	zinc finger protein 420
HGNC:2067	PDLIM1	PDZ and LIM domain 1
HGNC:20672	PHF8	PHD finger protein 8
HGNC:20690	ZNF425	zinc finger protein 425
HGNC:20710	ZBED2	zinc finger BED-type containing 2
HGNC:20711	ZBED3	zinc finger BED-type containing 3
HGNC:20712	ZDHHC18	zinc finger DHHC-type palmitoyltransferase 18
HGNC:20713	ZDHHC19	zinc finger DHHC-type palmitoyltransferase 19
HGNC:20714	ZDHHC16	zinc finger DHHC-type palmitoyltransferase 16
HGNC:20721	ZBED4	zinc finger BED-type containing 4
HGNC:20725	ZNF426	zinc finger protein 426
HGNC:20738	SYVN1	synoviolin 1
HGNC:20748	FANCL	FA complementation group L
HGNC:20749	ZDHHC20	zinc finger DHHC-type palmitoyltransferase 20

HGNC:20750	ZDHHC21	zinc finger DHHC-type palmitoyltransferase 21
HGNC:20751	WDFY3	WD repeat and FYVE domain containing 3
HGNC:20756	ZFYVE16	zinc finger FYVE-type containing 16
HGNC:20757	PLEKHF2	pleckstrin homology and FYVE domain containing 2
HGNC:20758	ZFYVE19	zinc finger FYVE-type containing 19
HGNC:20759	RBSN	rabenosyn, RAB effector
HGNC:20760	ZFYVE21	zinc finger FYVE-type containing 21
HGNC:20761	ZFYVE26	zinc finger FYVE-type containing 26
HGNC:20763	ANKFY1	ankyrin repeat and FYVE domain containing 1
HGNC:20764	PLEKHF1	pleckstrin homology and FYVE domain containing 1
HGNC:20767	PDLIM3	PDZ and LIM domain 3
HGNC:20804	ZNF428	zinc finger protein 428
HGNC:20807	ZNF486	zinc finger protein 486
HGNC:20808	ZNF430	zinc finger protein 430
HGNC:20809	ZNF431	zinc finger protein 431
HGNC:20810	ZNF432	zinc finger protein 432
HGNC:20811	ZNF433	zinc finger protein 433
HGNC:20812	ZSCAN32	zinc finger and SCAN domain containing 32
HGNC:20813	ZSCAN16	zinc finger and SCAN domain containing 16
HGNC:20814	ZNF436	zinc finger protein 436
HGNC:20816	PHF12	PHD finger protein 12
HGNC:20817	ZNF429	zinc finger protein 429
HGNC:20853	RNF145	ring finger protein 145
HGNC:20854	THAP2	THAP domain containing 2
HGNC:20855	THAP3	THAP domain containing 3
HGNC:20856	THAP1	THAP domain containing 1
HGNC:20868	ZBTB2	zinc finger and BTB domain containing 2
HGNC:20873	ZNF439	zinc finger protein 439
HGNC:20874	ZNF440	zinc finger protein 440
HGNC:20875	ZNF441	zinc finger protein 441
HGNC:20877	ZNF442	zinc finger protein 442
HGNC:20878	ZNF443	zinc finger protein 443
HGNC:20908	DZIP1	DAZ interacting zinc finger protein 1
HGNC:20964	WTIP	WT1 interacting protein
HGNC:20982	SMYD2	SET and MYND domain containing 2
HGNC:20986	SMYD1	SET and MYND domain containing 1
HGNC:20987	ANKMY1	ankyrin repeat and MYND domain containing 1
HGNC:20994	ZSCAN2	zinc finger and SCAN domain containing 2
HGNC:20997	ZMYND15	zinc finger MYND-type containing 15
HGNC:21000	MSS51	MSS51 mitochondrial translational activator
HGNC:21018	ZNF445	zinc finger protein 445
HGNC:21029	ZNF438	zinc finger protein 438
HGNC:21036	ZNF446	zinc finger protein 446
HGNC:21037	ZSCAN18	zinc finger and SCAN domain containing 18
HGNC:21039	ZNF449	zinc finger protein 449
HGNC:21067	SMYD4	SET and MYND domain containing 4
HGNC:21070	RNF121	ring finger protein 121
HGNC:21086	MIB1	MIB E3 ubiquitin protein ligase 1

HGNC:21091	ZNF451	zinc finger protein 451
HGNC:21143	ZBTB24	zinc finger and BTB domain containing 24
HGNC:21146	ZMYND19	zinc finger MYND-type containing 19
HGNC:21147	RNF122	ring finger protein 122
HGNC:21148	RNF123	ring finger protein 123
HGNC:21149	PRR3	proline rich 3
HGNC:21150	RNF125	ring finger protein 125
HGNC:21151	RNF126	ring finger protein 126
HGNC:21152	LONRF3	LON peptidase N-terminal domain and ring finger 3
HGNC:21153	RNF128	ring finger protein 128
HGNC:21154	RNF133	ring finger protein 133
HGNC:21155	MYLIP	myosin regulatory light chain interacting protein
HGNC:21156	PCGF6	polycomb group ring finger 6
HGNC:21158	RNF135	ring finger protein 135
HGNC:21159	RNF141	ring finger protein 141
HGNC:21161	TRIM68	tripartite motif containing 68
HGNC:21162	TRIM60	tripartite motif containing 60
HGNC:21174	ZC2HC1B	zinc finger C2HC-type containing 1B
HGNC:21175	ZC3H12D	zinc finger CCCH-type containing 12D
HGNC:21192	ZMYND12	zinc finger MYND-type containing 12
HGNC:21193	ZSCAN23	zinc finger and SCAN domain containing 23
HGNC:21200	ZNF454	zinc finger protein 454
HGNC:21224	ZUP1	zinc finger containing ubiquitin peptidase 1
HGNC:21225	CBLL1	Cbl proto-oncogene like 1
HGNC:21336	RNF146	ring finger protein 146
HGNC:21461	RC3H2	ring finger and CCCH-type domains 2
HGNC:21487	RNF217	ring finger protein 217
HGNC:21576	NHLRC1	NHL repeat containing E3 ubiquitin protein ligase 1
HGNC:21577	KDM1B	lysine demethylase 1B
HGNC:21578	RNF144B	ring finger protein 144B
HGNC:21628	ZNF460	zinc finger protein 460
HGNC:21629	ZNF461	zinc finger protein 461
HGNC:21653	TOPORS	TOP1 binding arginine/serine rich protein, E3 ubiquitin ligase
HGNC:21684	ZNF462	zinc finger protein 462
HGNC:21688	ZNHIT1	zinc finger HIT-type containing 1
HGNC:21698	RNF216	ring finger protein 216
HGNC:21711	ZNF804A	zinc finger protein 804A
HGNC:21734	LHX4	LIM homeobox 4
HGNC:21735	LHX6	LIM homeobox 6
HGNC:21740	FGD6	FYVE, RhoGEF and PH domain containing 6
HGNC:21767	ZNF853	zinc finger protein 853
HGNC:21806	ZNF786	zinc finger protein 786
HGNC:21894	PRKRIP1	PRKR interacting protein 1
HGNC:21919	PARP12	poly(ADP-ribose) polymerase family member 12
HGNC:21948	ZNF746	zinc finger protein 746
HGNC:21958	ZNF804B	zinc finger protein 804B
HGNC:21961	ZSCAN25	zinc finger and SCAN domain containing 25
HGNC:22043	ZNF713	zinc finger protein 713

HGNC:22048	NANOS3	nanos C2HC-type zinc finger 3
HGNC:22203	PHF14	PHD finger protein 14
HGNC:22213	ZNF777	zinc finger protein 777
HGNC:22220	ZNF470	zinc finger protein 470
HGNC:22224	KDM7A	lysine demethylase 7A
HGNC:22229	ZMIZ2	zinc finger MIZ-type containing 2
HGNC:22316	ZNRF2	zinc and ring finger 2
HGNC:2235	KLF6	Kruppel like factor 6
HGNC:22411	RNF148	ring finger protein 148
HGNC:22423	ZC3HAV1L	zinc finger CCCH-type containing, antiviral 1 like
HGNC:22785	ZNF727	zinc finger protein 727
HGNC:22788	FEZF1	FEZ family zinc finger 1
HGNC:22916	ZCCHC2	zinc finger CCHC-type containing 2
HGNC:22917	ZCCHC4	zinc finger CCHC-type containing 4
HGNC:22918	ZBTB3	zinc finger and BTB domain containing 3
HGNC:22958	PDLIM7	PDZ and LIM domain 7
HGNC:22982	JADE3	jade family PHD finger 3
HGNC:22983	PHF13	PHD finger protein 13
HGNC:22984	JADE2	jade family PHD finger 2
HGNC:22997	RTL3	retrotransposon Gag like 3
HGNC:23019	ZRSR2	zinc finger CCCH-type, RNA binding motif and serine/arginine rich 2
HGNC:23022	ZNF280B	zinc finger protein 280B
HGNC:23025	KLF14	Kruppel like factor 14
HGNC:23044	NANOS1	nanos C2HC-type zinc finger 1
HGNC:23137	RNF149	ring finger protein 149
HGNC:23138	RNF150	ring finger protein 150
HGNC:23154	ZNF467	zinc finger protein 467
HGNC:23179	ZNF761	zinc finger protein 761
HGNC:23187	THAP4	THAP domain containing 4
HGNC:23188	THAP5	THAP domain containing 5
HGNC:23189	THAP6	THAP domain containing 6
HGNC:23190	THAP7	THAP domain containing 7
HGNC:23191	THAP8	THAP domain containing 8
HGNC:23192	THAP9	THAP domain containing 9
HGNC:23193	THAP10	THAP domain containing 10
HGNC:23194	THAP11	THAP domain containing 11
HGNC:23216	ZNF469	zinc finger protein 469
HGNC:23226	ZNF471	zinc finger protein 471
HGNC:23235	RNF151	ring finger protein 151
HGNC:23239	ZNF473	zinc finger protein 473
HGNC:23241	ZFP62	ZFP62 zinc finger protein
HGNC:23245	ZNF474	zinc finger protein 474
HGNC:23258	ZNF479	zinc finger protein 479
HGNC:23272	ZNF805	zinc finger protein 805
HGNC:23279	DYTN	dystrotelin
HGNC:23292	NANOS2	nanos C2HC-type zinc finger 2
HGNC:23305	ZNF480	zinc finger protein 480
HGNC:23328	ZFP1	ZFP1 zinc finger protein

HGNC:23329	ZFP90	ZFP90 zinc finger protein
HGNC:23330	ZNF276	zinc finger protein 276
HGNC:23356	MARCHF8	membrane associated ring-CH-type finger 8
HGNC:23383	ZBTB26	zinc finger and BTB domain containing 26
HGNC:23384	ZNF483	zinc finger protein 483
HGNC:23385	ZNF484	zinc finger protein 484
HGNC:23386	MYCBP2	MYC binding protein 2
HGNC:23440	ZNF485	zinc finger protein 485
HGNC:2348	CREBBP	CREB binding protein
HGNC:23485	MORC4	MORC family CW-type zinc finger 4
HGNC:23486	ZCWPW1	zinc finger CW-type and PWWP domain containing 1
HGNC:23488	ZNF487	zinc finger protein 487
HGNC:23504	ZFAND4	zinc finger AN1-type containing 4
HGNC:23528	ZSWIM8	zinc finger SWIM-type containing 8
HGNC:23535	ZNF488	zinc finger protein 488
HGNC:23572	MORC3	MORC family CW-type zinc finger 3
HGNC:23573	MORC2	MORC family CW-type zinc finger 2
HGNC:23574	ZCWPW2	zinc finger CW-type and PWWP domain containing 2
HGNC:23589	ZNF503	zinc finger protein 503
HGNC:2360	CRIP1	cysteine rich protein 1
HGNC:2361	CRIP2	cysteine rich protein 2
HGNC:23640	ZNF322	zinc finger protein 322
HGNC:23705	ZNF490	zinc finger protein 490
HGNC:23706	ZNF491	zinc finger protein 491
HGNC:23707	ZNF492	zinc finger protein 492
HGNC:23708	ZNF493	zinc finger protein 493
HGNC:23709	ZSCAN4	zinc finger and SCAN domain containing 4
HGNC:23710	ZSCAN5A	zinc finger and SCAN domain containing 5A
HGNC:23712	ZSCAN1	zinc finger and SCAN domain containing 1
HGNC:23713	ZNF496	zinc finger protein 496
HGNC:23714	ZNF497	zinc finger protein 497
HGNC:23715	ZBTB45	zinc finger and BTB domain containing 45
HGNC:23716	ZNF500	zinc finger protein 500
HGNC:23717	ZNF501	zinc finger protein 501
HGNC:23718	ZNF502	zinc finger protein 502
HGNC:23721	ZC3HAV1	zinc finger CCCH-type containing, antiviral 1
HGNC:23780	ZNF506	zinc finger protein 506
HGNC:23783	ZNF507	zinc finger protein 507
HGNC:23784	MTA3	metastasis associated 1 family member 3
HGNC:23785	PIKFYVE	phosphoinositide kinase, FYVE-type zinc finger containing
HGNC:23803	ADNP2	ADNP homeobox 2
HGNC:23836	ZBTB5	zinc finger and BTB domain containing 5
HGNC:23847	ZBTB4	zinc finger and BTB domain containing 4
HGNC:23992	RTP4	receptor transporter protein 4
HGNC:24134	ZCCHC14	zinc finger CCHC-type containing 14
HGNC:24150	TRIM58	tripartite motif containing 58
HGNC:24156	PHF21A	PHD finger protein 21A
HGNC:24172	ZBTB8A	zinc finger and BTB domain containing 8A

HGNC:24277	ZC2HC1A	zinc finger C2HC-type containing 1A
HGNC:24280	PHF20L1	PHD finger protein 20 like 1
HGNC:24339	TRIM61	tripartite motif containing 61
HGNC:24343	CXXC1	CXXC finger protein 1
HGNC:24351	PHRF1	PHD and ring finger domains 1
HGNC:24457	DTX3	deltex E3 ubiquitin ligase 3
HGNC:24523	ZZZ3	zinc finger ZZ-type containing 3
HGNC:24544	RNF167	ring finger protein 167
HGNC:24566	PHF19	PHD finger protein 19
HGNC:24573	NEIL3	nei like DNA glycosylase 3
HGNC:24593	CXXC4	CXXC finger protein 4
HGNC:24605	ZNF521	zinc finger protein 521
HGNC:24636	LIMA1	LIM domain and actin binding 1
HGNC:24686	FBLIM1	filamin binding LIM protein 1
HGNC:2469	CSRP1	cysteine and glycine rich protein 1
HGNC:24693	MICAL2	microtubule associated monooxygenase, calponin and LIM domain containing 2
HGNC:24694	MICAL3	microtubule associated monooxygenase, calponin and LIM domain containing 3
HGNC:24699	SH3RF3	SH3 domain containing ring finger 3
HGNC:2470	CSRP2	cysteine and glycine rich protein 2
HGNC:24708	ZFP69	ZFP69 zinc finger protein
HGNC:2472	CSRP3	cysteine and glycine rich protein 3
HGNC:24722	ZNF614	zinc finger protein 614
HGNC:24737	ZNF569	zinc finger protein 569
HGNC:24740	ZNF615	zinc finger protein 615
HGNC:24750	ZNF699	zinc finger protein 699
HGNC:24751	ZNF792	zinc finger protein 792
HGNC:24762	ZC3H6	zinc finger CCCH-type containing 6
HGNC:24787	ZNF621	zinc finger protein 621
HGNC:24788	LONRF2	LON peptidase N-terminal domain and ring finger 2
HGNC:24804	RUFY4	RUN and FYVE domain containing 4
HGNC:24808	TRAFD1	TRAF-type zinc finger domain containing 1
HGNC:24819	ZBTB41	zinc finger and BTB domain containing 41
HGNC:24821	RFFL	ring finger and FYVE like domain containing E3 ubiquitin protein ligase
HGNC:24931	ZC4H2	zinc finger C4H2-type containing
HGNC:24992	ZNF706	zinc finger protein 706
HGNC:25000	ZNF571	zinc finger protein 571
HGNC:25001	ZBTB44	zinc finger and BTB domain containing 44
HGNC:25017	ZNF581	zinc finger protein 581
HGNC:25067	INTS12	integrator complex subunit 12
HGNC:25091	ZC3H18	zinc finger CCCH-type containing 18
HGNC:25092	ZNF765	zinc finger protein 765
HGNC:25105	SP140L	SP140 nuclear body protein like
HGNC:25108	ZNF551	zinc finger protein 551
HGNC:25112	ZNF845	zinc finger protein 845
HGNC:25135	LRSAM1	leucine rich repeat and sterile alpha motif containing 1
HGNC:25139	MARCHF9	membrane associated ring-CH-type finger 9
HGNC:25161	PHF21B	PHD finger protein 21B
HGNC:25162	NEURL3	neuralized E3 ubiquitin protein ligase 3

HGNC:25164	ZNF837	zinc finger protein 837
HGNC:25173	ZNF689	zinc finger protein 689
HGNC:25196	ZNF653	zinc finger protein 653
HGNC:25206	ZFAND2B	zinc finger AN1-type containing 2B
HGNC:25214	RTL4	retrotransposon Gag like 4
HGNC:25226	ZNF658	zinc finger protein 658
HGNC:25249	ZRANB3	zinc finger RANBP2-type containing 3
HGNC:25265	ZCCHC8	zinc finger CCHC-type containing 8
HGNC:25281	ZNF543	zinc finger protein 543
HGNC:25292	ZNF700	zinc finger protein 700
HGNC:25294	ZNF541	zinc finger protein 541
HGNC:25297	MEX3B	mex-3 RNA binding family member B
HGNC:25300	FBXL19	F-box and leucine rich repeat protein 19
HGNC:25321	SHARPIN	SHANK associated RH domain interactor
HGNC:25331	ZNF540	zinc finger protein 540
HGNC:25335	RNF214	ring finger protein 214
HGNC:25352	ZNF710	zinc finger protein 710
HGNC:25358	RNF170	ring finger protein 170
HGNC:25360	DDX59	DEAD-box helicase 59
HGNC:25370	ANKMY2	ankyrin repeat and MYND domain containing 2
HGNC:25392	ZNF568	zinc finger protein 568
HGNC:25404	FLYWCH1	FLYWCH-type zinc finger 1
HGNC:25406	ZNF664	zinc finger protein 664
HGNC:25420	RNF208	ring finger protein 208
HGNC:25424	ZCCHC9	zinc finger CCHC-type containing 9
HGNC:25503	RBM22	RNA binding motif protein 22
HGNC:25527	ANKZF1	ankyrin repeat and zinc finger peptidyl tRNA hydrolase 1
HGNC:25539	RFWD3	ring finger and WD repeat domain 3
HGNC:25544	MSL2	MSL complex subunit 2
HGNC:25552	RNF220	ring finger protein 220
HGNC:25574	TRIM62	tripartite motif containing 62
HGNC:25597	ZNF701	zinc finger protein 701
HGNC:25612	ZNF654	zinc finger protein 654
HGNC:25654	ZGRF1	zinc finger GRF-type containing 1
HGNC:25659	ZC3H11B	zinc finger CCCH-type containing 11B
HGNC:25669	ZNF556	zinc finger protein 556
HGNC:25677	ZKSCAN2	zinc finger with KRAB and SCAN domains 2
HGNC:25704	ZSWIM4	zinc finger SWIM-type containing 4
HGNC:25736	ZNF669	zinc finger protein 669
HGNC:25741	ZNF649	zinc finger protein 649
HGNC:25762	MUL1	mitochondrial E3 ubiquitin protein ligase 1
HGNC:25817	TUT7	terminal uridylyl transferase 7
HGNC:25821	ZNF668	zinc finger protein 668
HGNC:25827	ZNF613	zinc finger protein 613
HGNC:25843	ZNF750	zinc finger protein 750
HGNC:25844	ZMAT4	zinc finger matrin-type 4
HGNC:25858	ZFAND1	zinc finger AN1-type containing 1
HGNC:25872	ZNF696	zinc finger protein 696

HGNC:25879	ZNF606	zinc finger protein 606
HGNC:25883	ZNF703	zinc finger protein 703
HGNC:25885	ZNF665	zinc finger protein 665
HGNC:25893	ZC3H10	zinc finger CCCH-type containing 10
HGNC:25894	ZNF514	zinc finger protein 514
HGNC:25905	RNFT2	ring finger protein, transmembrane 2
HGNC:25917	FIZ1	FLT3 interacting zinc finger 1
HGNC:25919	ZNF566	zinc finger protein 566
HGNC:25932	ZNF844	zinc finger protein 844
HGNC:25949	ZNF586	zinc finger protein 586
HGNC:25950	ZNF562	zinc finger protein 562
HGNC:25953	ZNF280D	zinc finger protein 280D
HGNC:25954	ZCCHC10	zinc finger CCHC-type containing 10
HGNC:25955	ZNF280C	zinc finger protein 280C
HGNC:25959	GIN1	gypsy retrotransposon integrase 1
HGNC:25978	RNF186	ring finger protein 186
HGNC:26002	CASZ1	castor zinc finger 1
HGNC:26007	KRBOX4	KRAB box domain containing 4
HGNC:26021	LYAR	Ly1 antibody reactive
HGNC:26025	MARCHF5	membrane associated ring-CH-type finger 5
HGNC:26049	ZNF692	zinc finger protein 692
HGNC:26061	ZNF770	zinc finger protein 770
HGNC:26077	MARCHF1	membrane associated ring-CH-type finger 1
HGNC:26089	ZNHIT6	zinc finger HIT-type containing 6
HGNC:26135	ZNF552	zinc finger protein 552
HGNC:26138	ZFP2	ZFP2 zinc finger protein
HGNC:26166	ZNF574	zinc finger protein 574
HGNC:26179	ZNF672	zinc finger protein 672
HGNC:26191	ZNF385D	zinc finger protein 385D
HGNC:26209	ZCCHC7	zinc finger CCHC-type containing 7
HGNC:26245	ZBBX	zinc finger B-box domain containing
HGNC:26253	ZMYM1	zinc finger MYM-type containing 1
HGNC:26259	ZC3H12A	zinc finger CCCH-type containing 12A
HGNC:26273	ZNF768	zinc finger protein 768
HGNC:26279	ZNF671	zinc finger protein 671
HGNC:26299	SH3RF2	SH3 domain containing ring finger 2
HGNC:26302	LONRF1	LON peptidase N-terminal domain and ring finger 1
HGNC:26305	ZNF585A	zinc finger protein 585A
HGNC:26332	ZNF385B	zinc finger protein 385B
HGNC:26337	ZNF534	zinc finger protein 534
HGNC:26371	CBLL2	Cbl proto-oncogene like 2
HGNC:26408	ZNF599	zinc finger protein 599
HGNC:26416	ZNF570	zinc finger protein 570
HGNC:26420	ZNF573	zinc finger protein 573
HGNC:26421	ZNF582	zinc finger protein 582
HGNC:26422	ZNF558	zinc finger protein 558
HGNC:26427	ZNF583	zinc finger protein 583
HGNC:26432	ZNF547	zinc finger protein 547

HGNC:26433	ZMAT2	zinc finger matrin-type 2
HGNC:26449	ZNF578	zinc finger protein 578
HGNC:26457	ZNF681	zinc finger protein 681
HGNC:26479	ZNF778	zinc finger protein 778
HGNC:26484	ZNF560	zinc finger protein 560
HGNC:26496	ZNF785	zinc finger protein 785
HGNC:26498	ZNF513	zinc finger protein 513
HGNC:26513	NSMCE2	NSE2 (MMS21) homolog, SMC5-SMC6 complex SUMO ligase
HGNC:26539	DCST1	DC-STAMP domain containing 1
HGNC:26551	DZIP1L	DAZ interacting zinc finger protein 1 like
HGNC:26559	ZFYVE27	zinc finger FYVE-type containing 27
HGNC:26561	ZNF548	zinc finger protein 548
HGNC:26573	ZNF597	zinc finger protein 597
HGNC:26585	RTP5	receptor transporter protein 5 (putative)
HGNC:26629	ZNF554	zinc finger protein 554
HGNC:26632	ZNF549	zinc finger protein 549
HGNC:26636	ZBTB38	zinc finger and BTB domain containing 38
HGNC:26638	SWSAP1	SWIM-type zinc finger 7 associated protein 1
HGNC:26646	ZNF579	zinc finger protein 579
HGNC:26655	MARCHF10	membrane associated ring-CH-type finger 10
HGNC:26661	RNF168	ring finger protein 168
HGNC:26673	ZSCAN29	zinc finger and SCAN domain containing 29
HGNC:26687	ZNF610	zinc finger protein 610
HGNC:26698	TRIML1	tripartite motif family like 1
HGNC:26720	ZNF660	zinc finger protein 660
HGNC:26726	ZNF565	zinc finger protein 565
HGNC:26745	ZNF781	zinc finger protein 781
HGNC:26758	ZNF572	zinc finger protein 572
HGNC:26765	ZNF776	zinc finger protein 776
HGNC:26783	RNF185	ring finger protein 185
HGNC:26786	ZFP41	ZFP41 zinc finger protein
HGNC:2680	DIDO1	death inducer-obliterator 1
HGNC:26811	RNF152	ring finger protein 152
HGNC:26886	RNF19B	ring finger protein 19B
HGNC:26889	ZNF718	zinc finger protein 718
HGNC:26895	ZNF791	zinc finger protein 791
HGNC:26897	ZNF680	zinc finger protein 680
HGNC:26910	ZNF619	zinc finger protein 619
HGNC:26911	ZCCHC24	zinc finger CCHC-type containing 24
HGNC:26943	CXXC5	CXXC finger protein 5
HGNC:26955	ZBTB47	zinc finger and BTB domain containing 47
HGNC:26961	RNF169	ring finger protein 169
HGNC:26987	ZNF720	zinc finger protein 720
HGNC:26993	ZSWIM7	zinc finger SWIM-type containing 7
HGNC:26995	ZNF816	zinc finger protein 816
HGNC:26998	ZNF787	zinc finger protein 787
HGNC:270	PARP1	poly(ADP-ribose) polymerase 1
HGNC:27124	ZNF714	zinc finger protein 714

HGNC:27146	RNF187	ring finger protein 187
HGNC:27193	ZNF827	zinc finger protein 827
HGNC:27196	ZNF595	zinc finger protein 595
HGNC:27222	ZNF783	zinc finger family member 783
HGNC:27260	ZNF846	zinc finger protein 846
HGNC:27267	ZNF800	zinc finger protein 800
HGNC:27268	ZNF596	zinc finger protein 596
HGNC:27271	ZNF883	zinc finger protein 883
HGNC:27273	ZCCHC12	zinc finger CCHC-type containing 12
HGNC:27316	TRIM65	tripartite motif containing 65
HGNC:27318	ZNF584	zinc finger protein 584
HGNC:27387	ZDHHC24	zinc finger DHHC-type containing 24
HGNC:27465	ZNF740	zinc finger protein 740
HGNC:2752	PIAS1	protein inhibitor of activated STAT 1
HGNC:27571	RNF227	ring finger protein 227
HGNC:27603	ZNF780A	zinc finger protein 780A
HGNC:27606	ZNF575	zinc finger protein 575
HGNC:27611	ZNF841	zinc finger protein 841
HGNC:27614	ZNF763	zinc finger protein 763
HGNC:27713	ZNF852	zinc finger protein 852
HGNC:27729	RNF212	ring finger protein 212
HGNC:27735	RNF175	ring finger protein 175
HGNC:27752	RNF180	ring finger protein 180
HGNC:27801	ZNF789	zinc finger protein 789
HGNC:27815	ZNF707	zinc finger protein 707
HGNC:27984	ZNF517	zinc finger protein 517
HGNC:27994	ZNF850	zinc finger protein 850
HGNC:28028	ZNF691	zinc finger protein 691
HGNC:28037	RNF181	ring finger protein 181
HGNC:28038	MARCHF2	membrane associated ring-CH-type finger 2
HGNC:28040	MEX3C	mex-3 RNA binding family member C
HGNC:28043	ZNF821	zinc finger protein 821
HGNC:28046	ZMAT5	zinc finger matrin-type 5
HGNC:28053	ZFP69B	ZFP69 zinc finger protein B
HGNC:28054	ZNF628	zinc finger protein 628
HGNC:28062	ZNF616	zinc finger protein 616
HGNC:28063	ZNF766	zinc finger protein 766
HGNC:28068	ZNF605	zinc finger protein 605
HGNC:28071	ZNF799	zinc finger protein 799
HGNC:28073	ZFAND2A	zinc finger AN1-type containing 2A
HGNC:28079	ZNF598	zinc finger protein 598, E3 ubiquitin ligase
HGNC:28142	LIMD2	LIM domain containing 2
HGNC:28160	ZXDC	ZXD family zinc finger C
HGNC:28167	ZNF670	zinc finger protein 670
HGNC:28192	ZNF607	zinc finger protein 607
HGNC:28197	ZNF559	zinc finger protein 559
HGNC:28200	ZNF764	zinc finger protein 764
HGNC:28264	PCGF5	polycomb group ring finger 5

HGNC:28291	ZNF830	zinc finger protein 830
HGNC:28313	TET3	tet methylcytosine dioxygenase 3
HGNC:28322	ZNF524	zinc finger protein 524
HGNC:28323	ZBTB9	zinc finger and BTB domain containing 9
HGNC:28328	ZFC3H1	zinc finger C3H1-type containing
HGNC:28350	ZNF747	zinc finger protein 747
HGNC:28357	ZNF576	zinc finger protein 576
HGNC:28365	ZBTB37	zinc finger and BTB domain containing 37
HGNC:28382	ZNF555	zinc finger protein 555
HGNC:28418	ZNF684	zinc finger protein 684
HGNC:28428	PHF23	PHD finger protein 23
HGNC:28445	ZNF511	zinc finger protein 511
HGNC:28449	ZFTA	zinc finger translocation associated
HGNC:28495	ZNF683	zinc finger protein 683
HGNC:28501	ZNF775	zinc finger protein 775
HGNC:28510	GLIS3	GLIS family zinc finger 3
HGNC:28522	RNF182	ring finger protein 182
HGNC:28580	RTP1	receptor transporter protein 1
HGNC:28632	ZNF557	zinc finger protein 557
HGNC:28643	ZNF550	zinc finger protein 550
HGNC:28650	ZNF679	zinc finger protein 679
HGNC:28652	ZNF678	zinc finger protein 678
HGNC:28654	ZDHHC23	zinc finger DHHC-type palmitoyltransferase 23
HGNC:28671	ZNF546	zinc finger protein 546
HGNC:28673	ZNF577	zinc finger protein 577
HGNC:28682	ZFP82	ZFP82 zinc finger protein
HGNC:28684	ZNF561	zinc finger protein 561
HGNC:28696	ZNF567	zinc finger protein 567
HGNC:28710	ZNF843	zinc finger protein 843
HGNC:28721	RNF183	ring finger protein 183
HGNC:28724	APLF	aprataxin and PNKP like factor
HGNC:28728	MARCHF3	membrane associated ring-CH-type finger 3
HGNC:28730	ZNF677	zinc finger protein 677
HGNC:28742	ZNF620	zinc finger protein 620
HGNC:28766	ZNF611	zinc finger protein 611
HGNC:28838	LHX8	LIM homeobox 8
HGNC:28842	RBM4B	RNA binding motif protein 4B
HGNC:28854	ZNF667	zinc finger protein 667
HGNC:28855	ZNF630	zinc finger protein 630
HGNC:28856	RNF166	ring finger protein 166
HGNC:28857	ZNF682	zinc finger protein 682
HGNC:28917	JAZF1	JAZF zinc finger 1
HGNC:28972	ZC3H3	zinc finger CCCH-type containing 3
HGNC:28981	TUT4	terminal uridylyl transferase 4
HGNC:28986	ZNF592	zinc finger protein 592
HGNC:28990	ZNF516	zinc finger protein 516
HGNC:29003	ZNF609	zinc finger protein 609
HGNC:29004	ZNF646	zinc finger protein 646

HGNC:29005	TRIM66	tripartite motif containing 66
HGNC:29008	ZNF629	zinc finger protein 629
HGNC:29009	ZNF518A	zinc finger protein 518A
HGNC:29014	ZBTB39	zinc finger and BTB domain containing 39
HGNC:29025	ZNF536	zinc finger protein 536
HGNC:29027	ZZEF1	zinc finger ZZ-type and EF-hand domain containing 1
HGNC:29034	ATMIN	ATM interactor
HGNC:29045	ZBTB40	zinc finger and BTB domain containing 40
HGNC:29084	ZNF623	zinc finger protein 623
HGNC:29093	ZC3H11A	zinc finger CCCH-type containing 11A
HGNC:29122	VPS8	VPS8 subunit of CORVET complex
HGNC:29132	ABLIM3	actin binding LIM protein family member 3
HGNC:29147	ZNF652	zinc finger protein 652
HGNC:29151	DTX4	deltex E3 ubiquitin ligase 4
HGNC:29161	ZNF510	zinc finger protein 510
HGNC:29180	PHF24	PHD finger protein 24
HGNC:29189	ZFR2	zinc finger RNA binding protein 2
HGNC:29191	LIMCH1	LIM and calponin homology domains 1
HGNC:29212	ZNF512B	zinc finger protein 512B
HGNC:29222	ZNF644	zinc finger protein 644
HGNC:29238	ZNF608	zinc finger protein 608
HGNC:29243	RBM27	RNA binding motif protein 27
HGNC:29254	ZNF624	zinc finger protein 624
HGNC:29269	MARCHF4	membrane associated ring-CH-type finger 4
HGNC:29271	ZNFX1	zinc finger NFX1-type containing 1
HGNC:29277	ZNF687	zinc finger protein 687
HGNC:2928	DMD	dystrophin
HGNC:29297	ZNF530	zinc finger protein 530
HGNC:29299	ZSWIM5	zinc finger SWIM-type containing 5
HGNC:29312	ZFP14	ZFP14 zinc finger protein
HGNC:29313	ZDBF2	zinc finger DBF-type containing 2
HGNC:29316	ZSWIM6	zinc finger SWIM-type containing 6
HGNC:29328	ZNF529	zinc finger protein 529
HGNC:29334	ZFYVE28	zinc finger FYVE-type containing 28
HGNC:29362	ZC3H12C	zinc finger CCCH-type containing 12C
HGNC:29365	ZNF518B	zinc finger protein 518B
HGNC:29369	UNK	unk zinc finger
HGNC:29377	ZMAT1	zinc finger matrin-type 1
HGNC:29380	ZNF512	zinc finger protein 512
HGNC:29384	ZNF528	zinc finger protein 528
HGNC:29385	ZNF527	zinc finger protein 527
HGNC:29392	ZNF594	zinc finger protein 594
HGNC:29402	RNF157	ring finger protein 157
HGNC:29415	ZNF526	zinc finger protein 526
HGNC:29416	ZNF618	zinc finger protein 618
HGNC:29420	RSPRY1	ring finger and SPRY domain containing 1
HGNC:29423	ZNF525	zinc finger protein 525
HGNC:29425	ZNF721	zinc finger protein 721

HGNC:29434	RC3H1	ring finger and CCCH-type domains 1
HGNC:29448	ZNF717	zinc finger protein 717
HGNC:29450	GLIS2	GLIS family zinc finger 2
HGNC:29473	ZNF580	zinc finger protein 580
HGNC:29484	TET1	tet methylcytosine dioxygenase 1
HGNC:29525	GLIS1	GLIS family zinc finger 1
HGNC:29528	ZC3H15	zinc finger CCCH-type containing 15
HGNC:29535	MTF2	metal response element binding transcription factor 2
HGNC:29555	ZFP30	ZFP30 zinc finger protein
HGNC:29620	ZCRB1	zinc finger CCHC-type and RNA binding motif containing 1
HGNC:29653	ZNF771	zinc finger protein 771
HGNC:29672	MICALL2	MICAL like 2
HGNC:2976	DNMT1	DNA methyltransferase 1
HGNC:29804	MICALL1	MICAL like 1
HGNC:29897	NSMCE1	NSE1 homolog, SMC5-SMC6 complex component
HGNC:29913	ZC3HC1	zinc finger C3HC-type containing 1
HGNC:29941	GATAD1	GATA zinc finger domain containing 1
HGNC:29983	ZMAT3	zinc finger matrin-type 3
HGNC:29989	GATAD2A	GATA zinc finger domain containing 2A
HGNC:30021	HELZ2	helicase with zinc finger 2
HGNC:30027	JADE1	jade family PHD finger 1
HGNC:30047	LIMS3	LIM zinc finger domain containing 3
HGNC:30164	ZFAND6	zinc finger AN1-type containing 6
HGNC:30206	RNFT1	ring finger protein, transmembrane 1
HGNC:30246	ZCCHC17	zinc finger CCHC-type containing 17
HGNC:30256	PYGO1	pygopus family PHD finger 1
HGNC:30257	PYGO2	pygopus family PHD finger 2
HGNC:30285	RUFY3	RUN and FYVE domain containing 3
HGNC:3032	DRP2	dystrophin related protein 2
HGNC:30323	DTX3L	deltex E3 ubiquitin ligase 3L
HGNC:30461	ZNF626	zinc finger protein 626
HGNC:30487	ZNF773	zinc finger protein 773
HGNC:30489	ZNF688	zinc finger protein 688
HGNC:30498	ZNF563	zinc finger protein 563
HGNC:30541	ERI2	ERI1 exoribonuclease family member 2
HGNC:30550	MARCHF6	membrane associated ring-CH-type finger 6
HGNC:30552	PDZRN4	PDZ domain containing ring finger 4
HGNC:3057	DTNA	dystrobrevin alpha
HGNC:30570	ZNF627	zinc finger protein 627
HGNC:30571	ZNF625	zinc finger protein 625
HGNC:30574	ZNF519	zinc finger protein 519
HGNC:30577	MIB2	MIB E3 ubiquitin protein ligase 2
HGNC:3058	DTNB	dystrobrevin beta
HGNC:3060	DTX1	deltex E3 ubiquitin ligase 1
HGNC:30681	TAB3	TGF-beta activated kinase 1 (MAP3K7) binding protein 3
HGNC:30690	SP9	Sp9 transcription factor
HGNC:30700	TSHZ3	teashirt zinc finger homeobox 3
HGNC:30764	TRAIP	TRAF interacting protein

HGNC:30768	ZNF675	zinc finger protein 675
HGNC:30778	GATAD2B	GATA zinc finger domain containing 2B
HGNC:30781	TADA2B	transcriptional adaptor 2B
HGNC:30803	ZBED5	zinc finger BED-type containing 5
HGNC:30804	ZBED8	zinc finger BED-type containing 8
HGNC:30834	TRIM59	tripartite motif containing 59
HGNC:30869	ZC3H7B	zinc finger CCCH-type containing 7B
HGNC:30899	ZNF655	zinc finger protein 655
HGNC:30917	WIZ	WIZ zinc finger
HGNC:30936	ZNF823	zinc finger protein 823
HGNC:30938	DZIP3	DAZ interacting zinc finger protein 3
HGNC:30939	ZFHX4	zinc finger homeobox 4
HGNC:30940	ZNF532	zinc finger protein 532
HGNC:30941	ZC3H8	zinc finger CCCH-type containing 8
HGNC:30943	ZNF593	zinc finger protein 593
HGNC:30946	ZNF233	zinc finger protein 233
HGNC:30948	ZNF585B	zinc finger protein 585B
HGNC:30949	ZFP42	ZFP42 zinc finger protein
HGNC:30950	ZNF639	zinc finger protein 639
HGNC:30951	ZNF600	zinc finger protein 600
HGNC:30953	ZBTB10	zinc finger and BTB domain containing 10
HGNC:30954	ZNF695	zinc finger protein 695
HGNC:30955	ZNF587	zinc finger protein 587
HGNC:30958	ZNF622	zinc finger protein 622
HGNC:30959	ZC3H7A	zinc finger CCCH-type containing 7A
HGNC:30988	BNC2	basonuclin 2
HGNC:30990	ZSWIM2	zinc finger SWIM-type containing 2
HGNC:31106	ZNF564	zinc finger protein 564
HGNC:3121	E4F1	E4F transcription factor 1
HGNC:31446	ZBTB34	zinc finger and BTB domain containing 34
HGNC:31684	YY2	YY2 transcription factor
HGNC:31696	RNF165	ring finger protein 165
HGNC:31700	ZBTB7C	zinc finger and BTB domain containing 7C
HGNC:31749	ZCCHC13	zinc finger CCHC-type containing 13
HGNC:31834	ZNF641	zinc finger protein 641
HGNC:3185	EEA1	early endosome antigen 1
HGNC:31859	TRIM67	tripartite motif containing 67
HGNC:31930	ZNF662	zinc finger protein 662
HGNC:32034	ZNF697	zinc finger protein 697
HGNC:32281	ZNF705A	zinc finger protein 705A
HGNC:32284	ZNF705B	zinc finger protein 705B
HGNC:32286	ZNF723	zinc finger protein 723
HGNC:32291	ZNF704	zinc finger protein 704
HGNC:3238	EGR1	early growth response 1
HGNC:3239	EGR2	early growth response 2
HGNC:3240	EGR3	early growth response 3
HGNC:3241	EGR4	early growth response 4
HGNC:32458	ZNF716	zinc finger protein 716

HGNC:32459	ZCCHC18	zinc finger CCHC-type containing 18
HGNC:32460	ZNF724	zinc finger protein 724
HGNC:32462	ZNF726	zinc finger protein 726
HGNC:32463	ZNF728	zinc finger protein 728
HGNC:32464	ZNF729	zinc finger protein 729
HGNC:32466	ZNF735	zinc finger protein 735
HGNC:32467	ZNF736	zinc finger protein 736
HGNC:32468	ZNF737	zinc finger protein 737
HGNC:32469	ZNF738	zinc finger protein 738
HGNC:32470	ZNF730	zinc finger protein 730
HGNC:32486	RTP2	receptor transporter protein 2
HGNC:32550	ZBTB42	zinc finger and BTB domain containing 42
HGNC:32669	TRIM71	tripartite motif containing 71
HGNC:32671	TRIM72	tripartite motif containing 72
HGNC:32673	NR1H5P	nuclear receptor subfamily 1 group H member 5, pseudogene
HGNC:32686	TRIM75P	tripartite motif containing 75, pseudogene
HGNC:32783	ZNF749	zinc finger protein 749
HGNC:32947	RNF207	ring finger protein 207
HGNC:32962	ZDHHC11B	zinc finger DHHC-type containing 11B
HGNC:33104	ZIK1	zinc finger protein interacting with K protein 1
HGNC:33105	ZNF468	zinc finger protein 468
HGNC:33106	ZNF772	zinc finger protein 772
HGNC:33107	ZNF324B	zinc finger protein 324B
HGNC:33108	ZNF774	zinc finger protein 774
HGNC:33109	ZNF780B	zinc finger protein 780B
HGNC:33110	ZNF782	zinc finger protein 782
HGNC:33111	ZNF784	zinc finger protein 784
HGNC:33114	ZNF790	zinc finger protein 790
HGNC:33115	ZNF793	zinc finger protein 793
HGNC:33202	ZNF705D	zinc finger protein 705D
HGNC:33203	ZNF705E	zinc finger protein 705E
HGNC:33204	ZNF705F	zinc finger protein 705F
HGNC:33228	ZNF806	zinc finger protein 806
HGNC:33230	ZNF808	zinc finger protein 808
HGNC:33257	ZNF813	zinc finger protein 813
HGNC:33258	ZNF814	zinc finger protein 814
HGNC:33264	RFPL4B	ret finger protein like 4B
HGNC:33273	ZBED6	zinc finger BED-type containing 6
HGNC:33434	RNF215	ring finger protein 215
HGNC:33482	MEX3A	mex-3 RNA binding family member A
HGNC:33517	ZSCAN30	zinc finger and SCAN domain containing 30
HGNC:33609	MARCHF11	membrane associated ring-CH-type finger 11
HGNC:33722	ZNF385C	zinc finger protein 385C
HGNC:3373	EP300	E1A binding protein p300
HGNC:33879	DNLZ	DNL-type zinc finger
HGNC:34032	ZNF829	zinc finger protein 829
HGNC:34228	TRIM77	tripartite motif containing 77
HGNC:34246	ZSCAN5B	zinc finger and SCAN domain containing 5B

HGNC:34294	ZSCAN5C	zinc finger and SCAN domain containing 5C
HGNC:34332	ZNF835	zinc finger protein 835
HGNC:34333	ZNF836	zinc finger protein 836
HGNC:34495	ZSWIM9	zinc finger SWIM-type containing 9
HGNC:34513	ZNF860	zinc finger protein 860
HGNC:34517	RNF222	ring finger protein 222
HGNC:34519	ZNF862	zinc finger protein 862
HGNC:3467	ESR1	estrogen receptor 1
HGNC:3468	ESR2	estrogen receptor 2
HGNC:3471	ESRRA	estrogen related receptor alpha
HGNC:3473	ESRRB	estrogen related receptor beta
HGNC:3474	ESRRG	estrogen related receptor gamma
HGNC:3498	MECOM	MDS1 and EVI1 complex locus
HGNC:3508	EWSR1	EWS RNA binding protein 1
HGNC:35422	NEURL1B	neuralized E3 ubiquitin protein ligase 1B
HGNC:3581	BPTF	bromodomain PHD finger transcription factor
HGNC:360	AIRE	autoimmune regulator
HGNC:3663	FGD1	FYVE, RhoGEF and PH domain containing 1
HGNC:3664	FGD2	FYVE, RhoGEF and PH domain containing 2
HGNC:3702	FHL1	four and a half LIM domains 1
HGNC:3703	FHL2	four and a half LIM domains 2
HGNC:3704	FHL3	four and a half LIM domains 3
HGNC:37057	ZBTB8B	zinc finger and BTB domain containing 8B
HGNC:37116	ZAR1L	zygote arrest 1 like
HGNC:37134	ZNF705G	zinc finger protein 705G
HGNC:37138	ZNF732	zinc finger protein 732
HGNC:37142	ZNF587B	zinc finger protein 587B
HGNC:37146	TRIM43B	tripartite motif containing 43B
HGNC:37147	TRIM64B	tripartite motif containing 64B
HGNC:37148	TRIM64C	tripartite motif containing 64C
HGNC:37217	TRIM49D2	tripartite motif containing 49D2
HGNC:37245	ZGLP1	zinc finger GATA like protein 1
HGNC:37246	ZNF878	zinc finger protein 878
HGNC:37249	ZNF880	zinc finger protein 880
HGNC:37273	ZNF879	zinc finger protein 879
HGNC:38695	ZNF888	zinc finger protein 888
HGNC:38705	ZNF865	zinc finger protein 865
HGNC:38709	ZNF891	zinc finger protein 891
HGNC:38877	TRIM49C	tripartite motif containing 49C
HGNC:39941	LIMS4	LIM zinc finger domain containing 4
HGNC:40020	RNF223	ring finger protein 223
HGNC:4010	FUS	FUS RNA binding protein
HGNC:4170	GATA1	GATA binding protein 1
HGNC:4171	GATA2	GATA binding protein 2
HGNC:4172	GATA3	GATA binding protein 3
HGNC:4173	GATA4	GATA binding protein 4
HGNC:4174	GATA6	GATA binding protein 6
HGNC:41912	RNF224	ring finger protein 224

HGNC:4237	GFI1	growth factor independent 1 transcriptional repressor
HGNC:4238	GFI1B	growth factor independent 1B transcriptional repressor
HGNC:42955	TRIM49B	tripartite motif containing 49B
HGNC:4317	GLI1	GLI family zinc finger 1
HGNC:4318	GLI2	GLI family zinc finger 2
HGNC:4319	GLI3	GLI family zinc finger 3
HGNC:4320	GLI4	GLI family zinc finger 4
HGNC:43973	TRIM49D1	tripartite motif containing 49D1
HGNC:447	ZBED1	zinc finger BED-type containing 1
HGNC:45147	RFPL4AL1	ret finger protein like 4A like 1
HGNC:463	AMFR	autocrine motility factor receptor
HGNC:4662	GTF3A	general transcription factor IIIA
HGNC:4868	HERC2	HECT and RLD domain containing E3 ubiquitin protein ligase 2
HGNC:4897	HGS	hepatocyte growth factor-regulated tyrosine kinase substrate
HGNC:4909	HIC1	HIC ZBTB transcriptional repressor 1
HGNC:4920	HIVEP1	HIVEP zinc finger 1
HGNC:4921	HIVEP2	HIVEP zinc finger 2
HGNC:4928	ZNF875	zinc finger protein 875
HGNC:4929	ZSCAN22	zinc finger and SCAN domain containing 22
HGNC:4930	ZBTB48	zinc finger and BTB domain containing 48
HGNC:5024	HNF4A	hepatocyte nuclear factor 4 alpha
HGNC:5026	HNF4G	hepatocyte nuclear factor 4 gamma
HGNC:51249	RNF225	ring finger protein 225
HGNC:5275	KAT5	lysine acetyltransferase 5
HGNC:5401	SP110	SP110 nuclear body protein
HGNC:5542	IGHMBP2	immunoglobulin mu DNA binding protein 2
HGNC:590	BIRC2	baculoviral IAP repeat containing 2
HGNC:591	BIRC3	baculoviral IAP repeat containing 3
HGNC:592	XIAP	X-linked inhibitor of apoptosis
HGNC:5961	IKBKG	inhibitor of nuclear factor kappa B kinase regulatory subunit gamma
HGNC:6062	ING1	inhibitor of growth family member 1
HGNC:6063	ING2	inhibitor of growth family member 2
HGNC:6132	ISL1	ISL LIM homeobox 1
HGNC:6345	KLF1	Kruppel like factor 1
HGNC:6346	KLF12	Kruppel like factor 12
HGNC:6347	KLF2	Kruppel like factor 2
HGNC:6348	KLF4	Kruppel like factor 4
HGNC:6349	KLF5	Kruppel like factor 5
HGNC:6350	KLF7	Kruppel like factor 7
HGNC:6351	KLF8	Kruppel like factor 8
HGNC:644	AR	androgen receptor
HGNC:6513	LASP1	LIM and SH3 protein 1
HGNC:6593	LHX1	LIM homeobox 1
HGNC:6594	LHX2	LIM homeobox 2
HGNC:6595	LHX3	LIM homeobox 3
HGNC:660	TRIM23	tripartite motif containing 23
HGNC:6600	LIG3	DNA ligase 3
HGNC:6612	LIMD1	LIM domain containing 1

HGNC:6613	LIMK1	LIM domain kinase 1
HGNC:6614	LIMK2	LIM domain kinase 2
HGNC:6616	LIMS1	LIM zinc finger domain containing 1
HGNC:6633	LMCD1	LIM and cysteine rich domains 1
HGNC:6641	LMO1	LIM domain only 1
HGNC:6642	LMO2	LIM domain only 2
HGNC:6643	LMO3	LIM domain only 3
HGNC:6644	LMO4	LIM domain only 4
HGNC:6645	PRICKLE3	prickle planar cell polarity protein 3
HGNC:6646	LMO7	LIM domain 7
HGNC:6653	LMX1A	LIM homeobox transcription factor 1 alpha
HGNC:6654	LMX1B	LIM homeobox transcription factor 1 beta
HGNC:6657	LNX1	ligand of numb-protein X 1
HGNC:6679	LPP	LIM domain containing preferred translocation partner in lipoma
HGNC:6746	NBR1	NBR1 autophagy cargo receptor
HGNC:6775	ZFYVE9	zinc finger FYVE-type containing 9
HGNC:6848	MAP3K1	mitogen-activated protein kinase kinase kinase 1
HGNC:6914	MAZ	MYC associated zinc finger protein
HGNC:6916	MBD1	methyl-CpG binding domain protein 1
HGNC:6923	MBNL1	muscleblind like splicing regulator 1
HGNC:6973	MDM2	MDM2 proto-oncogene
HGNC:6974	MDM4	MDM4 regulator of p53
HGNC:7095	MID1	midline 1
HGNC:7096	MID2	midline 2
HGNC:7112	MKRN1	makorin ring finger protein 1
HGNC:7113	MKRN2	makorin ring finger protein 2
HGNC:7114	MKRN3	makorin ring finger protein 3
HGNC:7132	KMT2A	lysine methyltransferase 2A
HGNC:7133	KMT2D	lysine methyltransferase 2D
HGNC:7138	MLLT6	MLLT6, PHD finger containing
HGNC:7181	MNAT1	MNAT1 component of CDK activating kinase
HGNC:7198	MORC1	MORC family CW-type zinc finger 1
HGNC:7410	MTA1	metastasis associated 1
HGNC:7411	MTA2	metastasis associated 1 family member 2
HGNC:744	ASH2L	ASH2 like, histone lysine methyltransferase complex subunit
HGNC:7451	MTMR3	myotubularin related protein 3
HGNC:7452	MTMR4	myotubularin related protein 4
HGNC:7523	TRIM37	tripartite motif containing 37
HGNC:7622	MYT1	myelin transcription factor 1
HGNC:7623	MYT1L	myelin transcription factor 1 like
HGNC:7761	NEURL1	neuralized E3 ubiquitin protein ligase 1
HGNC:777	ZFHX3	zinc finger homeobox 3
HGNC:78	ABLIM1	actin binding LIM protein 1
HGNC:7803	NFX1	nuclear transcription factor, X-box binding 1
HGNC:7880	CNOT4	CCR4-NOT transcription complex subunit 4
HGNC:7960	NR0B1	nuclear receptor subfamily 0 group B member 1
HGNC:7961	NR0B2	nuclear receptor subfamily 0 group B member 2
HGNC:7962	NR1D1	nuclear receptor subfamily 1 group D member 1

HGNC:7963	NR1D2	nuclear receptor subfamily 1 group D member 2
HGNC:7965	NR1H2	nuclear receptor subfamily 1 group H member 2
HGNC:7966	NR1H3	nuclear receptor subfamily 1 group H member 3
HGNC:7967	NR1H4	nuclear receptor subfamily 1 group H member 4
HGNC:7968	NR1I2	nuclear receptor subfamily 1 group I member 2
HGNC:7969	NR1I3	nuclear receptor subfamily 1 group I member 3
HGNC:7971	NR2C1	nuclear receptor subfamily 2 group C member 1
HGNC:7972	NR2C2	nuclear receptor subfamily 2 group C member 2
HGNC:7973	NR2E1	nuclear receptor subfamily 2 group E member 1
HGNC:7974	NR2E3	nuclear receptor subfamily 2 group E member 3
HGNC:7975	NR2F1	nuclear receptor subfamily 2 group F member 1
HGNC:7976	NR2F2	nuclear receptor subfamily 2 group F member 2
HGNC:7977	NR2F6	nuclear receptor subfamily 2 group F member 6
HGNC:7978	NR3C1	nuclear receptor subfamily 3 group C member 1
HGNC:7979	NR3C2	nuclear receptor subfamily 3 group C member 2
HGNC:7980	NR4A1	nuclear receptor subfamily 4 group A member 1
HGNC:7981	NR4A2	nuclear receptor subfamily 4 group A member 2
HGNC:7982	NR4A3	nuclear receptor subfamily 4 group A member 3
HGNC:7983	NR5A1	nuclear receptor subfamily 5 group A member 1
HGNC:7984	NR5A2	nuclear receptor subfamily 5 group A member 2
HGNC:7985	NR6A1	nuclear receptor subfamily 6 group A member 1
HGNC:7988	NRAP	nebulin related anchoring protein
HGNC:8062	NUP153	nucleoporin 153
HGNC:8111	OSR1	odd-skipped related transcription factor 1
HGNC:8525	OVOL1	ovo like transcriptional repressor 1
HGNC:8762	PDCD2	programmed cell death 2
HGNC:8826	PEG3	paternally expressed 3
HGNC:8851	PEX10	peroxisomal biogenesis factor 10
HGNC:8910	PGR	progesterone receptor
HGNC:8919	PHF1	PHD finger protein 1
HGNC:8920	PHF2	PHD finger protein 2
HGNC:8921	PHF3	PHD finger protein 3
HGNC:9045	PLAG1	PLAG1 zinc finger
HGNC:9046	PLAGL1	PLAG1 like zinc finger 1
HGNC:9047	PLAGL2	PLAG1 like zinc finger 2
HGNC:9113	PML	PML nuclear body scaffold
HGNC:9232	PPARA	peroxisome proliferator activated receptor alpha
HGNC:9235	PPARD	peroxisome proliferator activated receptor delta
HGNC:9236	PPARG	peroxisome proliferator activated receptor gamma
HGNC:9346	PRDM1	PR/SET domain 1
HGNC:9347	PRDM2	PR/SET domain 2
HGNC:9348	PRDM4	PR/SET domain 4
HGNC:9349	PRDM5	PR/SET domain 5
HGNC:9350	PRDM6	PR/SET domain 6
HGNC:9351	PRDM7	PR/SET domain 7
HGNC:9397	ZMYND8	zinc finger MYND-type containing 8
HGNC:9440	THAP12	THAP domain containing 12
HGNC:952	BARD1	BRCA1 associated RING domain 1

HGNC:960	BAZ1A	bromodomain adjacent to zinc finger domain 1A
HGNC:961	BAZ1B	bromodomain adjacent to zinc finger domain 1B
HGNC:962	BAZ2A	bromodomain adjacent to zinc finger domain 2A
HGNC:963	BAZ2B	bromodomain adjacent to zinc finger domain 2B
HGNC:9717	PEX2	peroxisomal biogenesis factor 2
HGNC:9718	PXN	paxillin
HGNC:9831	RAG1	recombination activating 1
HGNC:9848	RANBP2	RAN binding protein 2
HGNC:9863	RAPSN	receptor associated protein of the synapse
HGNC:9864	RARA	retinoic acid receptor alpha
HGNC:9865	RARB	retinoic acid receptor beta
HGNC:9866	RARG	retinoic acid receptor gamma
HGNC:9886	KDM5A	lysine demethylase 5A
HGNC:9889	RBBP6	RB binding protein 6, ubiquitin ligase
HGNC:9896	RBM10	RNA binding motif protein 10
HGNC:9901	RBM4	RNA binding motif protein 4
HGNC:9902	RBM5	RNA binding motif protein 5
HGNC:9928	RBX1	ring-box 1
HGNC:9964	DPF2	double PHD fingers 2
HGNC:9965	RERE	arginine-glutamic acid dipeptide repeats
HGNC:9975	TRIM27	tripartite motif containing 27
HGNC:9976	TRIM13	tripartite motif containing 13
HGNC:9977	RFPL1	ret finger protein like 1
HGNC:9979	RFPL2	ret finger protein like 2
HGNC:9980	RFPL3	ret finger protein like 3

Supplementary Table S2. Primer sequences.

Name	F-sequence	R-sequence
DTX2	5'-CTGCGGGACCATCCTCATAG-3'	5'-CTCTTCCAGGCCACCTTCAG-3'
EGR2	5'-TTGACCAGATGAACGGAGTG-3'	5'-GCCCATGTAAGTGAAGGTCTG-3'
GATA2	5'-ATGAATGGGCAGAACCGACC-3'	5'-ATTTGCACAACAGGTGCCG-3'
SALL1	5'-CCTTCTCCTCATCGAGTGCC-3'	5'-GTCGTGCAGGGGTGCTATT-3'
ZEB2	5'-CACACAAGCCAGGGACAGAT-3'	5'-CTCGTAAGGTTTTTCACCACTGT-3'
ZNF93	5'-ACCTGACTGCCAAAACATGC-3'	5'-AGGTTGCTGGGTCTGCACTA-3'
ZNF146	5'-GCCAGACTTCCTGTGTTAACTCCT-3'	5'-CTGGTGCCTCTCTATCTGTGCATC-3'
GAPDH	5'-GCACCGTCAAGGCTGAGAAC-3'	5'-TGGTGAAGACGCCAGTGGA-3'
ZSCAN16	5'-TTCTTCCTAAAGACCTGCAAGC-3'	5'-TGTCCATGAGTATGTCCCGTC-3'
